# Dysregulated ceramides metabolism by fatty acid 2-hydroxylase exposes a metabolic vulnerability to target cancer metastasis

**DOI:** 10.1038/s41392-022-01199-1

**Published:** 2022-10-24

**Authors:** Xuantong Zhou, Furong Huang, Gang Ma, Wenqing Wei, Nan Wu, Zhihua Liu

**Affiliations:** 1 State Key Lab of Molecular Oncology, National Cancer Center/National Clinical Research Center for Cancer/Cancer Hospital, Chinese Academy of Medical Sciences and Peking Union Medical College, 100021 Beijing, China; 2 Key Laboratory of Carcinogenesis and Translational Research (Ministry of Education/Beijing), Department of Thoracic Surgery II, Peking University Cancer Hospital & Institute, 100142 Beijing, China; 3 Department of Gastric Surgery, Tianjin Medical University Cancer Institute and Hospital, National Clinical Research Center for Cancer; Key Laboratory of Cancer Prevention and Therapy, Tianjin; Tianjin’s Clinical Research Center for Cancer, 300060 Tianjin, P. R. China

**Keywords:** Drug development, Cancer metabolism, Metastasis, Gastrointestinal cancer, Oncogenes

## Abstract

Whereas it is appreciated that cancer cells rewire lipid metabolism to survive and propagate, the roles of lipid metabolism in metastasis remain largely unknown. In this study, using esophageal squamous cell carcinoma (ESCC) as a pulmonary metastasis model, we find that the enzyme fatty acid 2-hydroxylase (FA2H), which catalyzes the hydroxylation of free fatty acids (FAs), is enriched in a subpopulation of ESCC cells with high metastatic potential, and that FA2H knockdown markedly mitigates metastatic lesions. Moreover, increased FA2H expression is positively associated with poor survival in patients with ESCC. Lipidomics analysis identifies that two dihydroceramides—Cer(d18:0/24:0) and Cer(d18:0/24:1)—are increased in FA2H-depleted metastasizing ESCC cells. Upon administration, Cer(d18:0/24:0) and Cer(d18:0/24:1) impair the formation of overt metastases in a mouse experimental metastasis model. Then, forkhead box protein C2 (FOXC2) and FA2H are found to be co-upregulated in metastatic ESCC cell populations and ESCC specimens, and FA2H expression is further experimentally verified to be transcriptionally induced by FOXC2, which is boosted per se by tumour necrosis factor α (TNFα), a critical pro-metastasis cytokine in the tumour microenvironment, in metastasizing cells. Together, these results demonstrate that TNFα-FOXC2-FA2H is a novel signaling axis to promote metastasis, and its downstream dihydroceramide products could be promising drugs to intervene in metastasis.

## Introduction

Metastasis is the leading cause of cancer-related mortality. As metastasis involves diverse hostile microenvironments, these disseminated cells exploit various mechanisms to survive en route to and thrive in a distant organ.^1,2^ An emerging mechanism is that cancer cells efficiently adjust their metabolic programs during a harsh metastatic cascade.^3^ Several critical metabolites, such as lactate, pyruvate, glutamine, serine, alanine, proline, as well as asparagine, have been identified to play pro-metastasis roles in distinct cancer settings.^3^

Since metastases are of metabolic dependence, intervening in the functions of dysfunctional proteins in critical metabolic processes could prevent and/or treat metastasis in cell-line-derived or patient-derived xenograft models.^3^ Recent studies reported that monocarboxylate transporter 1 (MCT1), which transports extracellular lactate into the tumour cells, increased melanoma cells in circulation to promote the formation of metastatic lesions.^4^ When MCT1 inhibitor AZD3965 was administrated in mouse, and patient-derived melanoma models, the metastatic potential of melanoma cells was markedly impaired without affecting subcutaneous growth at primary sites.^4^ Despite the growing evidence highlighting the unique metabolic properties in metastatic cancer cells, the links between metabolism and metastatic formation are not yet fully understood.

Lipids are generally classified as fatty acids (FAs), triacylglycerols, glycerophospholipids, sphingolipids, and steroids, which are essential for building bio-membranes, storing energy, and relaying signals, respectively. Dysfunctional lipid metabolism in tumour initiation and progression has drawn growing attention in recent years.^5,6^ Key enzymes in uptake, de novo synthesis, and catabolic metabolism of lipids were aberrantly overexpressed or hyperactivated in distinct cancer types, accordingly sustaining growth, invasion, and metastasis in neoplastic cells.^5,6^ CD36 (also known as FAs translocase) delivers polyunsaturated fatty acids into cells, and its increased expression was indicative of poor prognosis in patients with cancers.^5^ Mechanistically, CD36 was enriched in a population of metastasis-initiating cells, and high-fat diets promoted the metastatic potential of CD36^high^ cells.^7^ Intriguingly, the neutralizing antibody blocking CD36 remarkably mitigated metastasis in melanoma and breast cancer.^7^ With respect to the biogenesis of FAs, adenosine triphosphate citrate lyase (ACLY), acetyl-CoA carboxylase (ACC), and fatty acid synthase (FASN) are three critical enzymes catalyzing the production of lipids, and their pro-metastasis functions were reported in multiple cancer settings.^6^ Three phase II clinical trials are currently in progress to evaluate the FASN inhibitor TVB-2640, as a single agent, in non-small cell lung cancer harboring KRAS mutations (NCT03808558), or synergistic with paclitaxel and trastuzumab to treat patients with advanced HER2^+^ breast cancer (NCT03179904), as well as combinatorial administration of TVB-2640 and bevacizumab in patients with first relapsed astrocytoma (NCT03032484).^5^ The catabolism of lipids was also involved in metastasis. Monoacylglycerol lipase (MAGL), which catalyzes the lysis of monoacylglycerols (MGs) to free FAs, was demonstrated to accelerate aggressive cellular traits, including metastasis. The catalytic site of MAGL was irreversibly blocked by JZL184, leading to the impairment of metastatic potential in lung, breast, and prostate cancer cells.^8–10^ These discoveries underline that metastatic cancer cells heavily rely on specific intracellular lipids, thereby prompting us to further investigate how rewired lipid metabolism contributes to metastasis.

As the lung is susceptible to metastatic lesions, we established two populations of cells with high potential for pulmonary metastasis—namely K30LM3 and K450LM2, respectively—from esophageal squamous cell carcinoma (ESCC) cell line KYSE30 and KYSE450 (named after K30P and K450P, respectively). Among the differentially expressed genes between the parental (K30P and K450P) and the metastasizing (K30LM3 and K450LM2) cells, the fatty acid 2-hydroxylase (FA2H) displayed increased expression in the latter populations. FA2H is responsible for adding a hydroxyl group to the C-2 position of free FAs to yield 2-hydroxy FAs, the products of which are further converted to a subset of sphingolipids and glycosphingolipids.^11,12^ FA2H and its catalytic products play vital roles in the epidermis and nervous system under physiological conditions, as exemplified by the observation that mutations in FA2H are implicated in spastic paraparesis and leukodystrophy.^13^ In recent years, it is becoming increasingly evident that FA2H also functions in several cancer types. FA2H was implicated in cAMP-induced cell cycle withdrawal in Schwannoma cell line D6P2T.^14^ Silencing of *FA2H* accelerated proliferation in gastric, colorectal, and breast cancer cells.^15–17^ Additionally, increased FA2H attenuated the resistance to cisplatin treatment in gastric cancer cells and rendered human cancer cells less refractory to antitumor drug PM02734.^15,18^ Moreover, FA2H is highly expressed in lung adenocarcinoma tissues compared with squamous and neuroendocrine carcinoma tissues, thereby leading to the accumulation of 2-hydroxy HexCers.^19^ FA2H was also reported to promote the migration in breast cancer cell line MDA-MB-231 and MCF-7 in vitro.^20^ These results indicate that FA2H is probably a key enzyme controlling the survival of cancer cells; however, the functions and the regulatory mechanisms of FA2H in cancer biology, especially in metastasis, remain largely unexplored. Hereafter, we investigated how FA2H regulated ESCC metastasis and identified TNFα activated FOXC2-FA2H axis as a novel signal axis to promote metastasis of ESCC cells.

## Results

### ESCC cells with high metastatic potential are enriched via in vivo selection

**Fig. 1:**
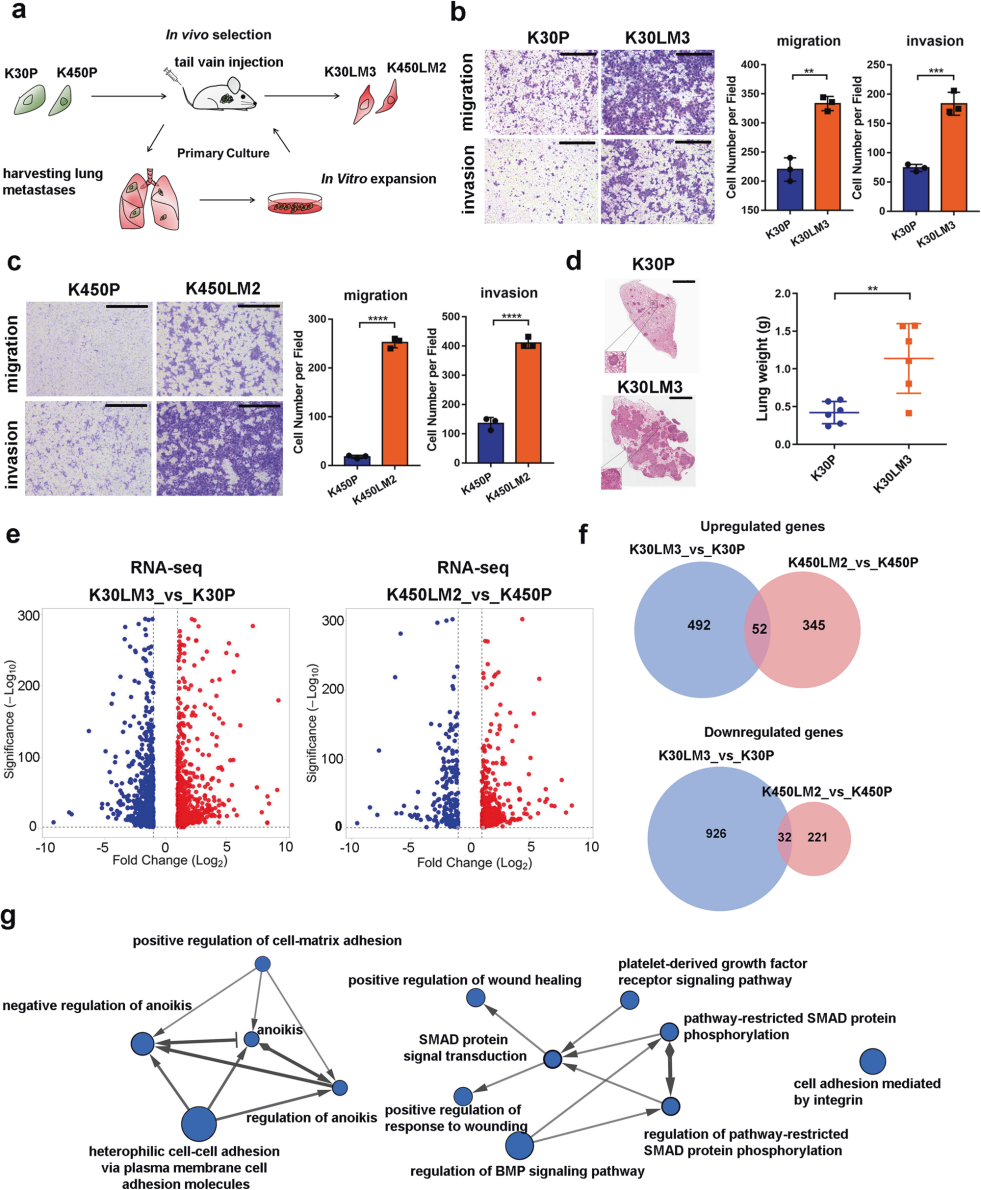
The selection of ESCC cells with high metastatic potential. **a** Schematic showing establishment of highly lung metastatic ESCC sublines by in vivo selection. **b**, **c** Transwell assay was performed to compare the migration and invasion ability of K30P/K30LM3 cells (**b**) or K450P/ K450LM2 cells (**c**). Scale bar, 500 µm. **d** Representative H&E images of lung tissues harvested from K30P and K30LM3 tail-vain injected mice (left panel). Scale bar, 2 mm. Statistical analysis of lung weight in each group (right panel). **e** Volcano plot showing the differential expressed genes between K30P and K30LM3 (left panel) or K450P and K450LM2 (right panel) cells by RNA-seq analysis (false-discovery rate (FDR) < 0.05, fold-change > 2). **f** Venn diagrams show upregulated and downregulated genes in LM cells relative to parental cells. **g** Cytoscape analysis shows the enrichment of metastasis related pathways in LM cells. The node size is proportional to the number of genes for each pathway term. Error bars denote mean ± SD. **P < 0.01, ***P < 0.001, ****P < 0.0001

In vivo selection enabled the enrichment of cancer cell subpopulations with enhanced metastatic potential.^21^ Accordingly, we first exploited a similar strategy to select highly metastatic ESCC cell sublines. Briefly, luciferase-expressing parental KYSE30 (K30P) and KYSE450 (K450P) cells were injected into the tail veins of SCID/Beige mice, respectively. As shown in Fig. 1a, we obtained highly metastatic KYSE30 (K30LM3) and KYSE450 (K450LM2) cell populations upon completing in vivo selection and in vitro propagation. Notably, K30LM3 and K450LM2 cells displayed both enhanced migration and invasiveness compared to the parental cells (Fig. 1b, c and Supplementary Fig. 1a, b). We next compared the lung metastasis potential between LM cells and parental cells, finding that LM cells yielded more extensive pulmonary lesions (Fig. 1d and Supplementary Fig. 1c–e). Interestingly, pulmonary metastatic loci occurred within only one month in mice injected with LM cells, while it took ~3 months for parental populations to form overt metastases (Data not shown).

To elucidate molecular mechanisms governing metastatic spread of LM cells to the lung, we performed RNA-sequencing (RNA-seq) analysis to characterize DEGs between LM and parental cells, revealing remarkable genetic differences based on unsupervised hierarchical clustering analysis (Fig. 1e and Supplementary Fig. 2a). In total, K30LM3 and K450LM2 cells shared 52 upregulated and 32 downregulated genes, among which some genes were answerable for enhanced metastatic potential (Fig. 1f). Geneset enrichment analysis (GSEA) showed that a subset of DEGs in common were significantly enriched in EMT gene signature (Supplementary Fig. 2b). Quantitative RT-PCR (qRT-PCR) analysis validated the upregulation of *Slug*, *Twist*, *Snail*, and *Vimentin* in LM cells (Supplementary Fig. 2c). Moreover, Gene Ontology (GO) and genetic network analysis revealed that DEGs were significantly enriched in key pro-metastasis processes, including cell motility, resistance to anoikis, integrin-mediated cell adhesion and TGFβ signaling (Fig. 1g and Supplementary Fig. 2d). Together, we preferentially enriched the lung metastatic subpopulations harboring essential molecular traits required for metastasis.

### FA2H promotes ESCC metastasis

**Fig. 2:**
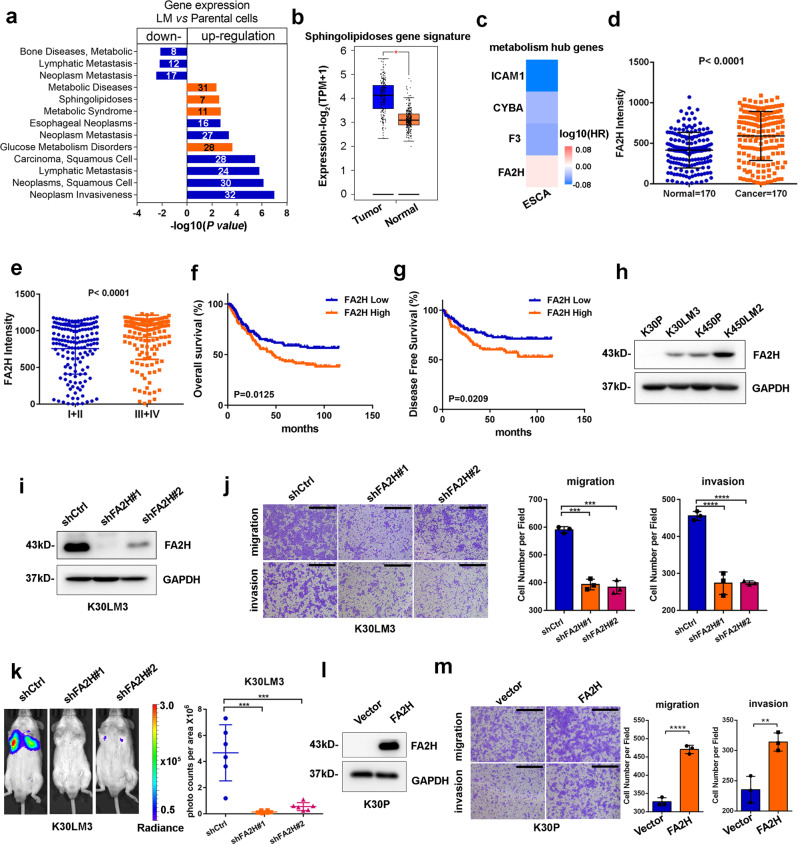
The function of FA2H in promoting ESCC metastasis. **a** Genomatix Disease Pathways analysis of co-regulated genes in LM cells relative to parental cells. **b** Sphingolipidoses gene signature is significantly enriched in TCGA Esophageal Cancer (ESCA) tumors. **c** A heatmap showing hazard ratios of disease-free survival between the top quarter and the bottom quarter of four metabolism-related hub genes expression in ESCA datasets. **d** Statistical analysis of FA2H staining intensity in 170 paired adjacent and ESCC tissues. **e** Statistical analysis of FA2H staining intensity between early-stage (I and II) and late-stage (III and IV) ESCC tumors (n = 306). **f**, **g** Kaplan–Meier analysis of overall survival (**f**) and disease-free survival (**g**) in ESCC patients with high or low FA2H expression (*n* = 306). The high or low expression of FA2H or FOXC2 in Kaplan–Meier analysis was stratified by median FA2H or FOXC2 protein levels from IHC staining quantifications. **h** Western blotting analysis of FA2H expression between LM cells and parental cells. **i** The FA2H knockdown efficiency was analyzed by western blotting in K30LM3 cells. **j** Transwell assay was performed to evaluate the effect of *FA2H* silencing on migration and invasion ability of K30LM3 cells. Scale bar, 500 µm. **k** Bioluminescence quantification of lung colonization by K30LM3 cells expressing shRNA targeting *FA2H* or a control hairpin (shCtrl). **l** Ectopic expression of FA2H in K30P cells was examined by Western blotting. **m** Transwell assay was performed to evaluate the effect of *FA2H* overexpression on migration and invasion ability of K30P cells. Scale bar, 500 µm. Error bars denote mean ± SD. *P < 0.05, **P < 0.01, ***P < 0.001, ****P < 0.0001

Intriguingly, disease pathway analysis highlighted that a subset of DEGs was significantly enriched in four metabolic gene signatures, such as “Sphingolipidoses” (Fig. 2a). We then analyzed these enriched metabolism-related gene signatures in The Cancer Genome Atlas (TCGA) esophageal cohort. Interestingly, only ‘Sphingolipidoses’ gene signature was markedly increased in tumour samples, suggesting sphingolipid metabolism may be associated with esophageal cancer progression (Fig. 2b and Supplementary Fig. 3a–c). The metabolism gene signature, consisting of four shared metabolic hub genes (i.e., *FA2H*, *ICAM1*, *F3*, and *CYBA*), was further identified to be remarkably elevated in TCGA esophageal cancer tissues compared with combined normal tissues from both TCGA and GTEx dataset. (Supplementary Fig. 3d, e). Importantly, the individual expression of *FA2H*, *ICAM1*, *F3,* and *CYBA* genes were further analyzed by retrieving ESCC samples or esophageal mucosa tissues from TCGA or GTEx datasets, respectively, confirming that their expressions were remarkably increased in ESCC compared with matched normal tissues (Supplementary Fig. 3f). Of note, cox hazards ratio analysis indicated higher expression of *FA2H* indicated an increased risk of esophageal cancer in the survival analysis than other three metabolic genes, strongly suggesting that predominant role of *FA2H* in esophageal cancer progression (Fig. 2c).

We further consolidated the clinical relevance of FA2H expression via immunohistochemistry (IHC) in a commercially available ESCC tissue microarray (#HEso-Squ150CS-02), containing ESCC (*n* = 170) and adjacent normal mucosa tissues (*n* = 170). The intensity of FA2H staining in ESCC tissues was markedly higher than that in adjacent normal tissues (Fig. 2d and Supplementary Fig. 3g). Notably, FA2H staining was increased when patients were diagnosed at advanced stages (III + IV vs. I + II) or with metastatic lesions (N > 0 vs. N = 0) (Fig. 2e and Supplementary Fig. 3h). Kaplan–Meier survival analysis further demonstrated that higher FA2H expression indicated unfavorable overall survival (OS) (*n* = 306, *P* = 0.0125; Fig. 2f and Supplementary Fig. 3i) and disease-free survival (DFS) (*n* = 306, *P* = 0.0209; Fig. 2g) in patients with ESCC.

In consistent with RNA-seq data, qRT-PCR showed that both the mRNA and protein level of FA2H were increased in LM cells, compared with parental cells (Fig. 2h and Supplementary Fig. 3j). By using lentiviruses encoding short hairpin RNAs (shRNAs), we depleted the expression of endogenous *FA2H* in LM cells, finding that *FA2H* depletion strikingly attenuated migration and invasion in vitro as well as lung metastasis in vivo (Fig. 2i–k and Supplementary Fig. 3k–n). Conversely, ectopically overexpression of FA2H in K30P cells significantly promoted cellular motility in vitro (Fig. 2l, m). Since metabolic enzymes display noncanonical functions in tumour initiation and progression,^22^ we asked if the pro-metastasis function of FA2H was dependent on its enzyme activity. We delivered FA2H^mut^ with reduced enzymatic activity or FA2H^wt^, respectively, into K30LM3 cells in the absence of endogenous *FA2H* (Supplementary Fig. 4a, b).^23^ We found that FA2H^mut^ markedly impaired the pro-motility capacity of this enzyme in cells, as compared with FA2H^wt^ (Supplementary Fig. 4c). Altogether, our data highlighted that *FA2H*, as an important gene in sphingolipidoses, exerted pro-metastasis function in ESCC.

### Silencing of *FA2H* reprograms ceramide metabolism to inhibit metastasis in LM cells

**Fig. 3:**
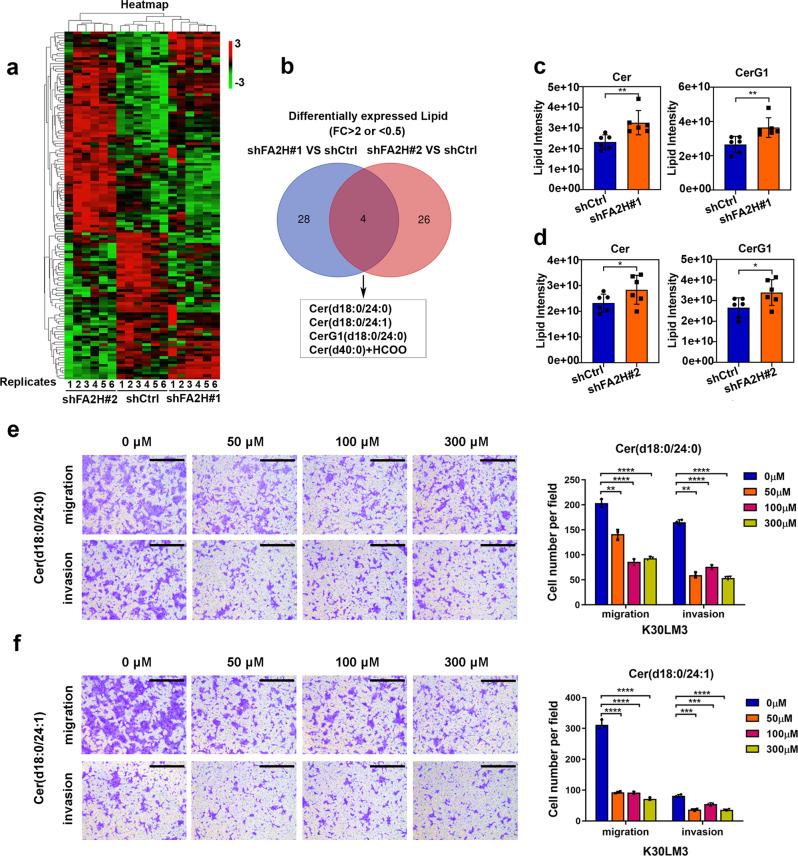
FA2H inhibits metastasis in LM cells by rewiring ceramide metabolism. **a** A heatmap showing the differential lipid species identified from lipidomics analysis in K30LM3 cells expressing two independent shRNA targeting *FA2H*, or a control hairpin (shCtrl). **b** Venn diagrams showing differential lipids identified in *FA2H*-depleted and control K30LM3 cells. **c**, **d** Two lipid species identified from K30LM3 expressing two independent shRNA targeting *FA2H*. **e** Transwell assay was performed to evaluate the effect of Cer (d18:0/24:0) on migration and invasion ability of K30LM3 cells. Scale bar, 500 µm. **f** Transwell assay was performed to evaluate the effect of Cer (d18:0/24:1) on migration and invasion ability of K30LM3 cells. Scale bar, 500 µm. Error bars denote mean ± SD. **P < 0.01, ***P < 0.001, ****P < 0.0001

It has been well-documented that FA2H catalyzes the biogenesis of 2-hydroxy FAs that are used to build hFA-sphingolipids and hFA-glycosphingolipids in mammalian cells.^13,24^ To investigate whether FA2H-catalyzed lipid metabolism plays a role in metastasis, we performed a lipidomic analysis in *FA2H*-depleted and control K30LM3 cells. A total of 2516 unique lipids, belonging to 34 lipid species, were identified through the integration of positive and negative electrospray ionization tandem MS, demonstrating that FA2H modulated the metabolic changes of downstream lipid species in K30LM3 cells (Fig. 3a and Supplementary Fig. 5a). Notably, *FA2H* ablation significantly increased the abundance of intracellular ceramides (Cer) and glucosylceramides (CerG1) on average, and three Cer and one CerG1 were simultaneously identified in two independently *FA2H*-depleted K30LM3 cell populations (FC > 2, *P* < 0.05) (Fig. 3b–d). Ceramide signaling has been shown to induce cell death through various mechanisms, yet its roles in metastasis remain less understood.^25^ As the levels of ceramides significantly increased in *FA2H*-depleted K30LM3 cells, it would be reasonable to speculate that specific ceramides were against the pro-metastasis function of FA2H. As expected, treatment of Cer (d18:0/24:0) or Cer (d18:0/24:1), two commercially available dihydroceramides, markedly impaired the motile potential and weakened the proliferation of K30LM3 cells (Fig. 3e, f and Supplementary Fig. 5b, c).

The desaturation of dihydroceramides to ceramides is mediated by dihydroceramide desaturase (DES).^26^ Pharmacological inhibition of DES activity with fenretinide has been demonstrated to increase the accumulation of endogenous dihydroceramides.^27^ Fenretinide has shown strong antitumor effects in various cancer settings and is currently tested in clinical trials.^28–30^ We supposed that fenretinide treatment phenocopied Cer (d18:0/24:0) or Cer (d18:0/24:1). As expected, fenretinide potently suppressed cell motility, and inhibited proliferation in a dose-dependent fashion (Supplementary Fig. 5d–f). Energy metabolism undergoes changes in cancer cells to support metastasis.^31,32^ We observed enhanced oxygen consumption rate (OCR) and extracellular acidification rate (ECAR) in LM cells, reflecting that metastasis required increasing energy input (Supplementary Fig. 5g–j). Additionally, It has been reported that ceramides species are implicated to modulate mitochondrial respiration and glycolysis in a variety of cell types.^33,34^ Interestingly, Cer (d18:0/24:0), Cer (d18:0/24:1), or fenretinide treatment markedly reduced OCR and ECAR levels relative to LM cells, respectively, suggesting dihydroceramides suppress the metastatic ability of LM cells by disrupting their dependency on energetic metabolism. These results suggested that FA2H-mediated ceramide metabolism conferred metastatic advantages to LM cells.

### Cer (d18:0/24:0) and Cer (d18:0/24:1) are promising agents to interrupt metastasis

**Fig. 4:**
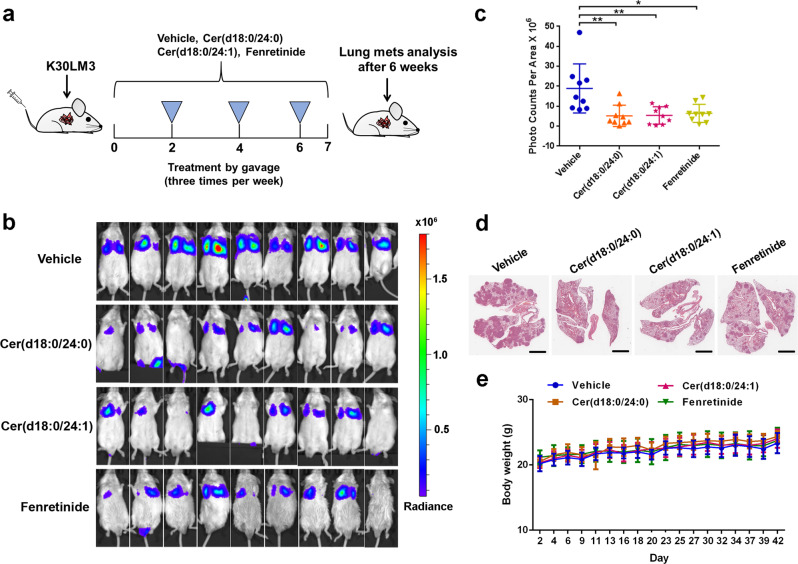
Cer (d18:0/24:0) and Cer (d18:0/24:1) interfere with metastasis. **a** Schematic representation showing details for therapeutic efficacy evaluation of Cer(d18:0/24:0), Cer(d18:0/24:1) or fenretinide treatment on an experimental lung metastasis model. **b** Bioluminescence quantification of lung colonization by K30LM3 treated with Cer(d18:0/24:0), Cer(d18:0/24:1), fenretinide or vehicle control. **c** Statistical analysis of bioluminescence quantification of lung colonization by K30LM3 in each treatment condition. **d** Representative H&E staining showing lung metastasis lesions in K30LM3 injected mice treated with Cer(d18:0/24:0), Cer(d18:0/24:1), fenretinide or vehicle control. Scale bar, 3 mm. **e** The effect of Cer(d18:0/24:0), Cer(d18:0/24:1), fenretinide or vehicle treatment on mice body weight was recorded. Error bars denote mean ± SD. *P < 0.05, **P < 0.01

Next, we employed an experimental lung metastatic model to test the translational value of Cer (d18:0/24:0) and Cer (d18:0/24:1). Upon injection of K30LM3 cells, male SCID/Beige mice were randomly divided into four groups. Each group was treated with Cer(d18:0/24:0), Cer(d18:0/24:1), fenretinide, and solvent (Vehicle group) by gavage, respectively. Intragastric administration was performed three times per week (Fig. 4a).^35^ As compared to the Vehicle group, Cer(d18:0/24:0), Cer(d18:0/24:1), or fenretinide treatment significantly attenuated lung metastases, as demonstrated by markedly reduced radiance intensity of luciferase and lung weights with metastatic lesions (Fig. 4b–d and Supplementary Fig. 6a). Importantly, mice from the treated groups were well-tolerated, as no weight loss and no significant histopathological changes in hearts, kidneys, and livers were observed during treatment course (Fig. 4e and Supplementary Fig. 6b). Together, these preclinical findings provided a novel therapeutic strategy to inhibit lung metastasis with low toxicity profile.

### *FA2H* is transcriptionally increased by FOXC2

**Fig. 5:**
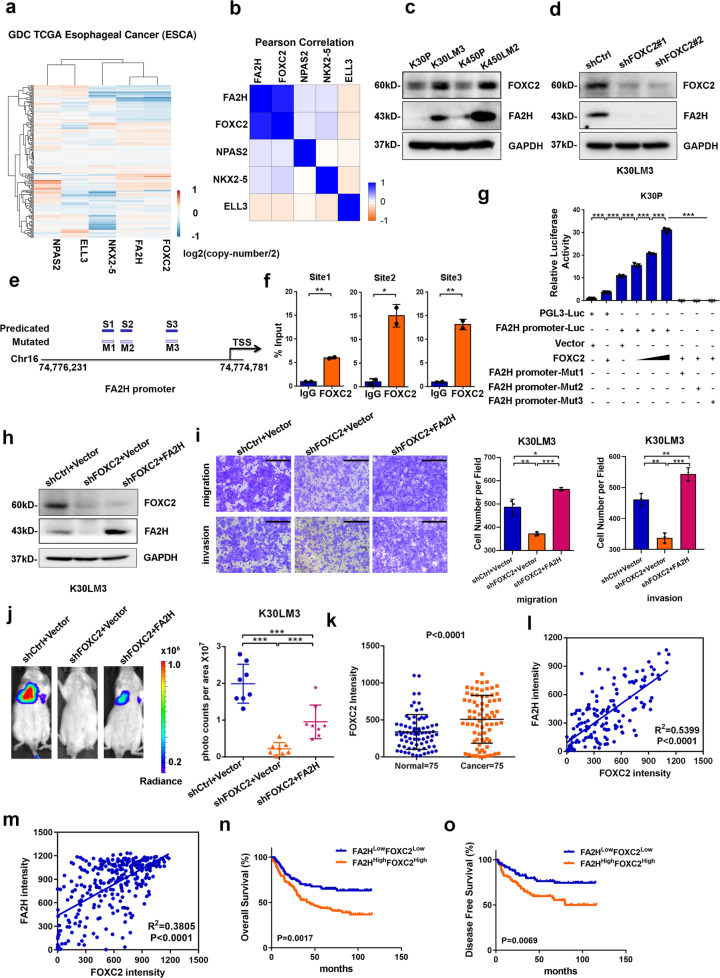
FOXC2 transcriptionally mediates *FA2H* expression to promote metastasis. **a** A heatmap showing of copy number variations of LM cells co-upregulated genes in ESCA dataset. **b** Pearson correlation analysis of copy number variations of LM cells co-upregulated genes in ESCA dataset. **c** Western blotting analysis of FOXC2, FA2H protein level between LM and parental cells. **d** The *FOXC2* knockdown efficiency was analyzed by western blotting in K30LM3 cells. **e** Schematic showing the putative FOXC2 binding regions on *FA2H* promoter. **f** Chromatin immunoprecipitation (ChIP) analysis of FOXC2 occupancy on *FA2H* promoters in K30LM3 cells. **g** Luciferase activity of the pGL3-luciferase vector (pGL3-Luc), *FA2H* promoter-luciferase (*FA2H* promoter-Luc) or mutant *FA2H* promoter-luciferase (*FA2H* promoter-Mut1/2/3) reporter gene in K30P cells transiently transfected with FOXC2 or vector, *n* = 3 wells per group. **h** Western blotting analysis of FOXC2, FA2H protein level in K30LM3 cells expressing a control hairpin (shCtrl), shRNA targeting *FOXC2*, shRNA targeting *FOXC2* with additional *FA2H* overexpression. **i** Transwell assay was performed to evaluate the effect of *FOXC2* silencing alone and *FOXC2* silencing with additional *FA2H* overexpression on migration and invasion ability of K30LM3 cells. Scale bar, 500 µm. **j** Bioluminescence quantification of lung colonization by K30LM3 expressing shRNA targeting *FOXC2*, shRNA targeting *FOXC2* with additional *FA2H* overexpression, or a control hairpin (shCtrl). **k** Statistical analysis of FOXC2 staining intensity in 75 paired adjacent and ESCC tissues. **l** Pearson correlation analysis of FOXC2 and FA2H staining intensity in 75 paired adjacent and ESCC tissues. **m** Pearson correlation analysis of FOXC2 and FA2H staining intensity in ESCC tissues (*n* = 306). **n, o** Kaplan–Meier analysis of overall survival (**n**) and disease-free survival (**o**) in ESCC patients with FA2H^Low^FOXC2^Low^ (*n* = 103) or FA2H^High^FOXC2^High^ (*n* = 103) expression. Error bars denote mean ± SD. *P < 0.05, **P < 0.01, ***P < 0.001

Transcription factors (TFs) play essential roles in tumour progression and metastasis, and individual TF has been identified in each step of the metastasis cascade.^36^ We thereby tried to identify the TF that directly regulated *FA2H* expression in this model, finding four TFs (FOXC2, NPAS2, NKX2-5, and ELL3) based on pathway analysis (Supplementary Fig. 2d). Among these TFs, the increased *FOXC2* expression was positively correlated with *FA2H* in both K30LM3 and K450LM2 cells relative to parental cells (Supplementary Fig. 7a, b). In addition, co-amplification of *FOXC2* and *FA2H* was observed in 185 patients with esophageal cancer from TCGA cohort, whereas no prominent correlation was found between *FA2H* and the other three TFs (Fig. 5a, b). These findings suggested that *FA2H* was positively associated with *FOXC2* at both genomic and transcriptional levels.

To ascertain FOXC2 regulated *FA2H* transcription, we first measured *FOXC2* and *FA2H* mRNA levels, showing that both were upregulated in LM cells relative to parental cells (Supplementary Fig. 7c, d). Consistently, the protein abundance of these two genes was also increased in two LM cells (Fig. 5c). Depletion of *FOXC2* dramatically decreased *FA2H* expression in both LM cells (Fig. 5d and Supplementary Fig. 7e–g). There are three putative binding sites of FOXC2 in *FA2H* promoter, as predicted from JASPAR database (Fig. 5e and Supplementary Fig. 7h, i). The occupancy of FOXC2 on *FA2H* promoter was then validated by chromatin immunoprecipitation (ChIP) assay (Fig. 5f and Supplementary Fig. 7j). Additionally, dual-luciferase reporter assay demonstrated that overexpression of *FOXC2* in parental cells remarkably enhanced the activity of *FA2H* promoter, and mutation in every single binding site of FOXC2 dramatically blunted the transcriptional activity of *FA2H* promoter (Fig. 5g and Supplementary Fig. 7k), suggesting that integrity of all the three *cis*-elements and coordination between each other were indispensable for FOXC2-mediated transactivation of *FA2H*.

Previous studies have reported that FOXC2 promotes cancer metastasis.^37,38^ In line with this, silencing of *FOXC2* in LM cells decreased migratory and invasive potential in vitro and lung metastasis in vivo (Supplementary Fig. 7l–n). We restored *FA2H* expression in stable *FOXC2*-depleted K30LM3 cells and evaluated the metastatic ability of K30LM3 cells. We found that *FA2H* reintroduction markedly attenuated the inhibitory effect of *FOXC2* depletion on the motility in vitro and the metastasis in vivo (Fig. 5h–j and Supplementary Fig. 8a–d). Conversely, knockdown of *FA2H* in *FOXC2*-overexpressing K30P cells weakened the pro-motility roles of FOXC2 in vitro (Supplementary Fig. 8e–g). Collectively, these data demonstrated that the regulation of *FA2H* level by FOXC2 is functionally significant during metastasis.

To investigate the clinical relevance of FOXC2-directed *FA2H* transcription in ESCC, we examined FOXC2 protein level in 75 pairs of ESCC and adjacent normal mucosa tissues, finding that FOXC2 protein was significantly increased in ESCC tissues and the staining intensity of FOXC2 was positively correlated with FA2H (Fig. 5k–m and Supplementary Fig. 8h). Higher FOXC2 expression was indicative of poorer survival in patients with ESCC (*n* = 306) (Supplementary Fig. 8i–k). More importantly, patients with concurrently high FOXC2 and FA2H expression had much worse OS and DFS (Fig. 5n, o).

### TNFα activates FOXC2-FA2H axis to boost metastasis

**Fig. 6:**
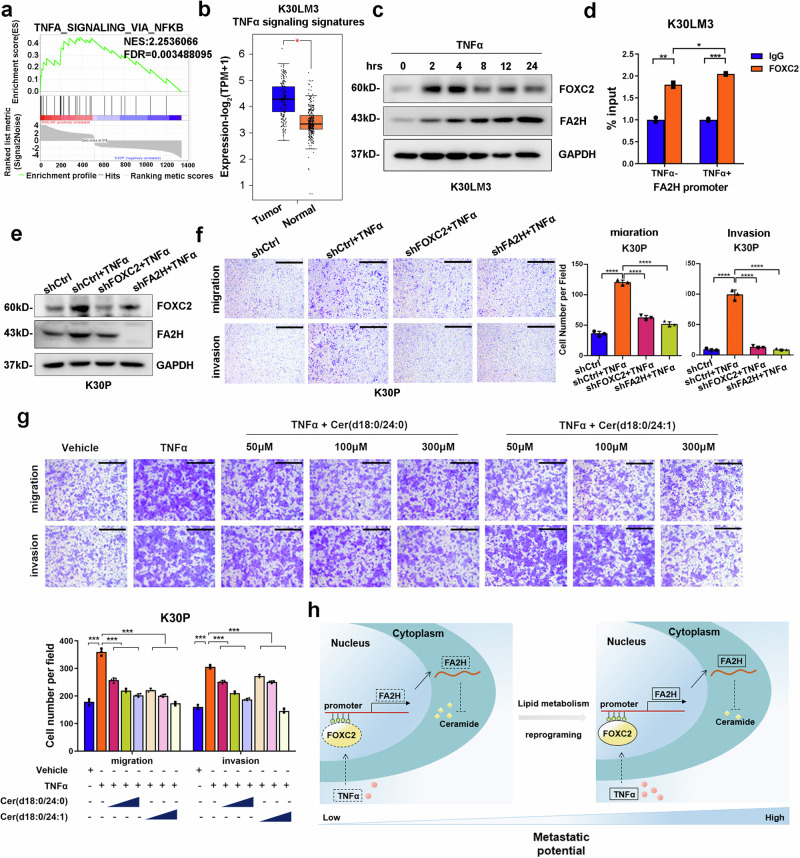
TNFα promotes metastasis by activating FOXC2-FA2H axis. **a** Geneset enrichment analysis shows ‘TNFα signaling via NF-κB’ gene signature were significantly enriched in K30LM3 relative to K30P cells. Each of the black bars represents a gene in the pathway. **b** Analysis of TNFα signaling gene signature identified from K30LM3 cells in TCGA ESCA dataset using GEPIA2 tool. **c** Western blotting analysis of FOXC2 and FA2H protein level after TNFα treatment at different time points in K30LM3 cells. **d** ChIP analysis of FOXC2 occupancy on the promoters of *FA2H* upon TNFα treatment. **e** Western blotting analysis of TNFα-induced FOXC2 and FA2H protein level in K30P expressing shRNA targeting *FOXC2, FA2H* or a control hairpin (shCtrl). **f** Transwell assay analysis of TNFα-induced *FOXC2* and *FA2H* expression on migration and invasion ability in K30P cells expressing shRNA targeting *FOXC2, FA2H* or a control hairpin (shCtrl). Scale bar, 500 µm. **g** Representative images of K30P transwell assay with TNFα treatment alone or in combination with Cer(d18:0/24:0) or Cer(d18:0/24:1) in a serial concentration. Scale bar, 500 µm. **h** Proposed working model for TNFα-activated FOXC2-FA2H axis governed ceramide metabolic reprogramming in ESCC. Error bars denote mean ± SD. *P < 0.05, **P < 0.01, ***P < 0.001, ****P < 0.0001

Extracellular factors are imperative to support the colonization and outgrowth of metastasizing tumour cells.^39^ Inflammatory cytokines or chemo-attractants have been reported to pre-determine and maintain metastasis in a variety of tumour models.^40,41^ In LM cells, GSEA analysis of DEGs showed that cytokine signaling, particularly TNFα-NF-κB signaling, was activated (Fig. 6a and Supplementary Fig. 9a–g), indicating that TNFα probably facilitated metastasis of LM cells. Additionally, these TNFα signaling signature genes were also ectopically overexpressed in TCGA esophageal cancer tissues (Fig. 6b and Supplementary Fig. 9h). Previous studies demonstrated that TNFα stimulated experimental pulmonary metastasis in melanoma and fibrosarcoma mouse models.^42,43^ Similarly, treatment of TNFα increased the migration and invasion in both LM and parental cells (Supplementary Fig. 9i, j).

TNFα promotes tumor progression through different mechanisms, including enhancing EMT, cell survival, and cancer stem cell (CSC) expansion.^41^ In differentiated 3T3-L1 adipocytes, TNFα induced FOXC2 expression via PI3K and ERK 1/2 signaling pathways;^44^ however, there is no evidence to demonstrate that TNFα directly regulates FOXC2 expression in cancer cells. One previous study showed that NF-κB enhanced lung cancer cell motility by inducing FOXC2 expression.^45^ Whereas TNFα activates NF-κB signaling pathway under most conditions, we thereby examined whether TNFα treatment affected FOXC2 level and subsequent *FA2H* transcription in ESCC, finding that TNFα elicited co-upregulation of FOXC2 and FA2H in both LM and parental cells (Fig. 6c and Supplementary Fig. 9k–r). The occupancy of FOXC2 on *FA2H* promoter was also increased upon TNFα treatment (Fig. 6d). Meanwhile, TNFα-activated FOXC2-FA2H axis was partially diminished by depleting *FOXC2 or FA2H*, leading to impaired motile potential in vitro (Fig. 6e, f and Supplementary Fig. 10a, b).

Finally, we sought to determine whether FA2H-catalyzed ceramide metabolism blocked TNFα-activated proliferation and invasion. TNFα conferred parental cells with higher proliferative and invasive ability, which was sufficiently abrogated by either Cer (d18:0/24:0) or Cer (d18:0/24:1) treatment in a dose-dependent fashion (Fig. 6g and Supplementary Fig. 10c–h). Taken together, these findings demonstrated that TNFα signaling governed metastasis of LM cells through activating FOXC2-FA2H axis, which resulted in dysregulated ceramide metabolism that can be potentially exploited as a novel and promising therapeutic strategy to intervene in such processes.

## Discussion

Metastasis is a tough journey, during which tumour cells confront various exogenous and endogenous insults. However, few metastasis-specific gene mutations have been identified in a pan-cancer analysis of metastatic tumors.^46^ Additionally, whereas aberrant alterations in oncogenes and/or tumor suppressor genes confer growth advantages to transformed cells in primary sites, they are not always sufficient to facilitate colonization in distal sites. An emerging paradigm is that tumor cells exploit metabolic machinery to respond to constantly changing microenvironments and boost final metastatic growth since metabolism is highly dynamic and is intertwined with (epi-)genetic landscape.^47,48^ In this study, a subset of DEGs between parental and LM populations are enriched in metabolic processes such as sphingolipidoses. Interestingly, sphingolipidoses gene signature is markedly increased in ESCA samples relative to normal esophageal tissues. FA2H, as a critical enzyme in sphingolipid metabolism, is highly expressed in LM populations and then defined to promote metastasis via loss/gain-of-function approaches. More importantly, FA2H is indicative of metastasis and poor outcome in patients with ESCC. Whereas FA2H plays the pro-metastasis role in ESCC cells, it is of note that FA2H functions in an opposite manner in gastric and colorectal cancer.^15,16^ FA2H was highly expressed in normal gastric and colorectal tissues, and (*R*)-2-hydroxy palmitic acid, one direct metabolite of FA2H, markedly mitigated the growth and invasiveness of gastric and colorectal cancer cells.^15,16^ On the other hand, significantly changed lipids from *FA2H*-depleted ESCC cells were distinct from those described in gastric and colorectal cancer cells with enforced FA2H expression. Thus, the various functions of FA2H may result from its expression and catalytic products, which are determined by distinct tissue contexts.

Ceramides, belonging to sphingolipids, cooperated with glycosylceramides and cholesterol to generate signaling platforms and influence tumour progression.^25^ Besides the well-defined roles of ceramides in cell death,^49^ its functions in metastasis have been uncovered recently. CerS4-derived ceramides inhibited metastasis by promoting the binding between TβRI and its inhibitor Smad7, hence preventing TβRI from localizing in primary cilia to activate the sonic hedgehog signaling pathway.^50^ In this study, Cer(d18:0/24:0) and Cer(d18:0/24:1), which were significantly increased in the *FA2H*-knockdown LM cells, markedly attenuated spread to the lung and suppressed growth to some extent. The anti-metastasis function of Cer(d18:0/24:1) is in accord with the most recent finding that it mitigated the motile potential of SKOV3 cells by impairing lamellipodia formation.^51^ However, Cer(d18:0/24:0) promoted proliferation and metastasis in gallbladder cancer via activating mTOR signaling pathway.^52^ These discrepant findings reflect that the functions of distinct ceramides are largely determined by their different lengths of acyl chains and diverse cancer settings.^25^ We also tested the translational value of Cer(d18:0/24:0) and Cer(d18:0/24:1) in an experimental metastasis model, showing that they reduced pulmonary metastasis without obviously damaging major organs of recipient nude mice. Therefore, Cer(d18:0/24:0) and Cer(d18:0/24:1) hold promises as novel anti-metastasis agents in ESCC.

We then found that FOXC2 knockdown transcriptionally reduced *FA2H* level and hence suppressed pulmonary metastases of K30LM3 and K450LM2 cells. Two major mechanisms whereby FOXC2 promotes metastasis have been unveiled. It has been reported FOXC2 elicited EMT via increasing ZEB1 expression.^53^ Additionally, FOXC2 also facilitated angiogenesis and lymphangiogenesis.^54,55^ Notably, FOXC2 is implicated in lipid metabolism, as FOXC2 sustained survival of preadipocytes via Akt/mTORC1 signaling pathway in mice and increased oxidation of FAs in mouse adipose.^56,57^ However, direct evidence supporting that FOXC2 assists metastasis via regulating lipid metabolism remains sparse. Here, we provide a paradigm to support a novel pro-metastasis mechanism of FOXC2.

Moreover, we explored environmental cues to increase FOXC2 expression, and TNFα came to the forefront largely because several DEGs were enriched in TNFα-NF-κB signaling pathway. As expected, administration of TNFα markedly increased FOXC2 expression and its subsequent binding to *FA2H* promoter. FOXC2 is likely to be a novel downstream effector of TNFα, as strongly suggested by our findings and the evidence that TNFα-NF-κB directly increased *FOXC2* transcription.^45^ This study highlights an emerging mechanism that TNFα promotes metastasis by modulating lipid metabolism. Together, these results demonstrate that TNFα-FOXC2-FA2H axis promotes lung metastasis in ESCC.

There are still some limitations to this study. First, we did not examine the role of each increased cytokine, such as IL6, in FOXC2/FA2H-mediated metastasis; instead, TNFα was used as a representative to show FOXC2 expression was upregulated in response to extracellular stimuli. In our opinion, regulation of FOXC2 expression could be multifactorial, thereby detailed clarification warrants further investigations. Second, we did not discriminate the anti-metastasis mechanisms of Cer(d18:0/24:0) from that of Cer(d18:0/24:1), and it is not clear how they blunted mitochondrial respiration. While Sun et al. showed a possible link between FA2H and glucose metabolism,^16^ this question is still open. Moreover, we did not track exactly each step of synthesis of Cer(d18:0/24:0) and Cer(d18:0/24:1), so it is not clear how these two dihydroceramides increased upon silencing of *FA2H*. De novo synthesis and salvage pathway function synergistically to maintain intracellular homeostasis of ceramide pool by producing and converting distinct ceramide species,^25^ thus increased levels of Cer(d18:0/24:0) and Cer(d18:0/24:1) are probably the outcome of complex interactions between the two pathways. Nevertheless, this study unveils that TNFα-FOXC2-FA2H axis rewires lipid metabolism to potentiate pulmonary metastasis, and nominates the potential of Cer(d18:0/24:0) and Cer(d18:0/24:1) as anti-metastasis drugs (Fig. 6h).

## Material and methods

### Cell culture

The human ESCC cell lines KYSE30 and KYSE450 were generously provided by Dr. Y. Shimada (Kyoto University, Kyoto, Japan) and maintained in RPMI 1640 supplemented with 10% fetal bovine serum (FBS) (HyClone, South Logan, UT, USA). HEK293T were purchased from the American Type Culture Collection (ATCC) (Manassas, VA, USA) and maintained in DMEM supplemented with 10% fetal bovine serum (FBS) (HyClone, South Logan, UT, USA). For in vivo selection and bioluminescent imaging, KYSE30 and KYSE450 cells were infected with lentivirus expressing firefly luciferase and selected with G418 (200 µg/mL) for two weeks.

### Antibodies and reagents

Antibodies used in this study were as follows: Anti-FOXC2 (PA524588, Invitrogen), Anti-FA2H (15452-1-AP, Proteintech), Anti-GAPDH (2118, Cell Signaling Technology). Secondary antibodies included HRP Goat Anti-Mouse (926-80010, LI-COR) and HRP Goat Anti-Rabbit (926-80011, LI-COR). Cer(d18:0/24:0) and Cer(d18:0/24:1) were purchased from Avanti Lipids Polar. Fenretinide was purchased from MedChemExpress (HY-15373), and TNFα was purchased from Novus (#210-TA).

### Animal study

All procedures and experimental protocols were approved by the Institutional Animal Care and Use Committee of Chinese Academy of Medical Sciences Cancer Hospital.

For the establishment of highly lung metastatic ESCC cell lines, in vivo selection was followed as previously described.^21^ Briefly, 1 × 10^6^ K30P and K450P cells were intravenously injected into SCID/Beige mice. For the first round selection, lung metastatic lesions were observed after 3 months for both cells. Mice were then sacrificed, and metastatic nodules were dissected and dissociated into pieces, and grown in the culture dishes. G418 were used to select and differentiate tumor cells from fibroblast and other stromal cells. These G418-resistant tumor cells were denoted as the first generation of lung metastatic derivatives (LM1). Similarly, LM1 cells were subjected to another round of in vivo selection by injecting them into the SCID/Beige mice, which gave rise to lung metastatic nodes and the second generation of lung metastatic derivatives (LM2). Accordingly, K30P or K450P were subjected to three or two rounds of in vivo selection, namely K30LM3 and K450LM2, respectively.

For experimental lung metastasis assay, 6-week-old male SCID/Beige mice were purchased from Vital River (Beijing, China). 1 × 10^6^ K30LM3 cells with stable *FOXC2/FA2H* depletion or *FOXC2*-depleted K30LM3 cells with additional *FA2H* overexpression and control cells were established and injected into the lateral tail veins of SCID/Beige mice. The pulmonary colonization of tumor cells was monitored by bioluminescent imaging after 40 days.

For dihydroceramides-based antimetastasis therapy, 1 × 10^6^ K30LM3 cells were first intravenously injected. After one week, vehicle, Cer(d18:0/24:0) or Cer(d18:0/24:1) was administered three times a week by oral gavage with a dose of 10.26 mg/kg/day. Fenretinide (120 mg/kg/day) were treated as positive control. Mice body weights in each group were measured during each treatment.

For bioluminescent imaging, mice were anesthetized and injected with 10 µL/g of D-luciferin (PerkinElmer) in DPBS intraperitoneally. After 15 min, bioluminescence (BLI) was captured via Xenogen Optical in vivo Imaging System (IVIS; Xenogen). BLI was normalized to a background value, which was defined from luciferin-injected mice without tumor cells.^21^

To evaluate the toxicity of Cer(d18:0/24:0), Cer(d18:0/24:1), and Fenretinide treatment, the mice organs, including hearts, livers, and kidneys, were harvested and paraffin-embedded followed by H&E staining. Histological examinations were performed to analyze the treatment-related morphological changes.

### Plasmids and lentivirus packaging

FOXC2 and FA2H cDNA clones were subcloned into the pLVX-puro plasmid. shRNA oligos targeting *FOXC2* or *FA2H* and a nontargeting oligo control were engineered into pSIH-puro plasmid. For pLVX-puro lentivirus production, the packaging plasmids Δ8.9, pLP2 were used. For pSIH-puro lentivirus production, the packaging plasmids vSVG, pLP1 and pLP2 were used. The indicated packaging plasmids and lentiviral vectors were co-transfected into HEK293T cells. After 48 h transfection, the supernatant containing lentivirus particles were collected and stored in aliquots at −80 °C. For lentivirus infection, cells were first treated with polybrene (5 µg/mL) (TR-1003, Sigma), then infected with the indicated lentivirus. Stable cell populations were established by selecting puromycin (2 μg/mL) (540222, Sigma) for 2 weeks.

### Transwell assay

Parental KYSE30 (6 × 10^4^ per insert) or KYSE450 cells (2 × 10^5^ per insert), as well as LM cells with indicated treatment, were suspended in FBS-free RPMI 1640 and seeded into the upper chambers with or without precoated matrigel (BD, Franklin Lakes, NJ, USA). The bottom chambers were added with RPMI 1640 medium supplemented with 10% FBS. After 24 h incubation, the migratory or invasive cells were methanol-fixed and stained with crystal violet. Cells in three randomly selected fields were photographed and statistically analyzed.

### RNA extraction and qRT-PCR

Total RNA was extracted with TRIzol reagent (Thermo Fisher Scientific). The cDNAs were obtained using Quantscript RT kit (Tiangen, Beijing, China) according to the manufacturer’s protocol. Real-time RT-PCR was performed by using SYBR Premix Ex TaqTM II (TaKaRa, Japan) on Step-one plus real-time PCR system (Applied Biosystems, Foster City, CA, USA), according to the manufacturer’s instructions. The primers used are listed in Supplementary Table 1.

### RNA-seq and data analysis

RNA-seq analysis was performed as previously described.^58^ For comparison of gene expression profiles between parental K30P, K450P cells, and metastatic K30LM3, K450LM2 cells, cells were collected with three biological replicated in each group. Total RNA was extracted with Trizol reagents (Thermo Fisher Scientific). For library preparation, Poly(A) + mRNA was isolated and enriched using NEBNext Poly(A) mRNA Magnetic Isolation Module. The mRNA was then recovered for library generation with NEBNext ® Ultra™ Directional RNA Library Prep Kit for Illumina (NEB, E7420S), following the manufacturer’s instructions. The cDNA libraries were sequenced at WuXiNextCODE. FastQC was used to examine the quality of the raw reads. Read alignment was conducted using STAR (v2.5.1b), R package edgeR (v3.8.5) was used to determine relative transcript abundances and differentially expressed genes (DEGs) between sample pair. The DEGs (Fold-change > 2-fold or 1.5, *P* < 0.05) are listed in Supplementary Table 2.

### Lipidomics analysis

Total lipids were extracted from 1 × 10^7^ *FA2H*-depleted K30LM3 cells and control cells with six biological replicates in each group.^59^ Briefly, cell pellets were mixed well with 200 µL ultra-pure water. Following the addition of 240 µL methanol to the mixture, samples were vortexed and added with MTBE, and vortexed again. After being placed at room temperature for 20 min, samples were centrifuged at 8000 × *g* for 15 min at 4 °C. The resulting lipid fraction was carefully collected and dried with nitrogen gas. Dried samples can be stored at −80 °C. Before LC/MS, the samples were redissolved with isopropanol, vortexed then centrifuged at 8000 × *g* for 15 min at 4 °C. The supernatant was then subjected to UHPLC Nexera LC-30A for analysis. LipidSearch software version 4.1(Thermo Scientific™) was applied for peak recognition, peak alignment and lipid characterization, and quantitative processing with the following parameters (5 ppm precursor tolerance, 5% product ion threshold, and deletion of lipid molecules with a RSD > 30%). LipidSearch data was further normalized by total peak area normalization, and lipid molecules with the missing value >50% in each group were deleted. After the process of Pareto-scaling, SIMPCA-P 14.1 (Umetrics, Umea, Sweden) software was applied for multivariate statistical analysis, including principal component analysis (PCA), partial least squares discriminant analysis (PLS-DA), as well as orthogonal partial least squares discriminant analysis (OPLS-DA). Identification of differentially expressed lipid species was based on the VIP (Variable Importance for the Projection) values from OPLS-DA model. The lipids with the threshold of VIP > 1, *P* < 0.05 in the univariate statistical analysis (Student’s *t*-test) were considered statistically significant. The results of Lipidomics were listed in Supplementary Table 3.

### Cell proliferation assay

Cell proliferation was quantified by CCK-8 assays. K30P, K450P, 30LM3, and 450LM2 with indicated treatment were seeded into 96-well plates (2 × 10^4^ cells/mL; 100 µL/well). Cell Proliferation Reagent CCK-8 (#CK04, Dojindo Molecular Technologies, Japan) was used for measuring cell proliferation. After 1 h of incubation at 37 °C, absorbance at 450 nm was measured using a microplate reader (BioTek).

### Immunohistochemistry assay

Tissue microarrays were stained with anti-FOXC2 (PA524588, Invitrogen) and anti-FA2H (15452-1-AP, Proteintech) antibodies. The representative images of IHC staining were captured by Aperio ScanScope (Leica, Nussloch, Germany).

### Western blotting

Western blot was performed according to the standard protocol. Briefly, cells were harvested and lysed in RIPA buffer (1% NP-40, 0.1% sodium dodecyl sulfate (SDS), 50 mM Tris–HCl pH 7.4, 150 mM NaCl, 0.5% sodium deoxycholate, 1 mM (EDTA), 1× proteinase inhibitor cocktail (Roche)) for 30 min on ice. The proteins were resolved on 10% SDS-PAGE and transferred onto PVDF membranes (Millipore). The membranes were blocked with 5% milk powder solution, then incubated with specific antibodies at 4 °C overnight. Following incubation with secondary antibodies, immunoblots were visualized using the ImageQuant LAS-4000 System (GE). Antibodies for western blotting are listed in ‘Antibodies and reagents’.

### ChIP assay

ChIP assay was performed as previously described.^60^ Briefly, indicated cells were crosslinked with 1% formaldehyde for 10 min at room temperature, and cell pellets were collected and subjected to sonication. The sheared chromatin was then diluted, followed by immune clearance for 1 h at 4 °C. Immunoprecipitation was performed by adding 4 µg of specific antibodies and incubated at 4 °C overnight. Next, protein A-Sepharose beads were added and incubated for at least 1 h with rotation. The beads were then washed sequentially for 10 min each in TSE I, TSE II, and buffer III and finally twice with TE buffer. Chromatin complexes were eluted with elution buffer (1% SDS, 0.1 M NaHCO3), and crosslinking was reversed at 65 °C overnight. DNA fragments were purified with the QIAquick PCR purification kit (28104, Qiagen) and used for quantitative PCR reactions with Power SYBR Green PCR Master Mix reagent (Applied Biosystems). Primers used for ChIP are listed in Supplementary Table 1.

### Seahorse metabolic assay

For all assays, cells were pretreated with 4 mg/mL BSA, 100 μM Cer(d18:0/24:0), 100 μM Cer(d18:0/24:1), and 2.5 μM fenretinide for 24 h and then 5 × 10^3^ treated K30LM3 or 6 × 10^3^ treated K450LM2 cells were seeded in XF96 cell culture plates incubated in a 5% CO_2_ incubator at 37 °C overnight.

For Seahorse XF Cell Mito Stress Test, OCR was measured using the Seahorse XF Cell Mito Stress Test Kit (103015-100, Agilent Technologies, America) on an XFe96 Analyzer. Final concentrations of 2.5 μM oligomycin, 2 μM Carbonyl cyanide-4 (trifluoromethoxy) phenylhydrazone (FCCP) and 0.5 μM Rotenone/antimycin A (Rot/AA) were used for all conditions. Basal respiration, maximal respiration, and ATP production were calculated as follows:

$$\begin{array}{l}{\mathrm{Basal}}\;{\mathrm{respiration}} = \left({\mathrm{last}}\;{\mathrm{rate}}\;{\mathrm{measurement}}\;{\mathrm{before}}\;{\mathrm{first}}\;{\mathrm{injection}}\right)\\ \qquad\qquad\qquad\qquad\quad- \,\left( {{{{\mathrm{non}} {\mbox{-}} {\mathrm{mitochondrial}}}}\;{{{\mathrm{respiration}}}}\;{{{\mathrm{rate}}}}} \right)\end{array}$$

$$\begin{array}{l}{{{\mathrm{Maximal}}}}\;{{{\mathrm{respiration}}}} = \left( {{{{\mathrm{maximum}}}}\;{{{\mathrm{rate}}}}\;{{{\mathrm{measurement}}}}\;{{{\mathrm{after}}}}\;{{{\mathrm{FCCP}}}}\;{{{\mathrm{injection}}}}} \right)\\ \qquad\qquad\qquad\qquad\qquad\, - \,\left( {{{{\mathrm{non}} {\mbox{-}} {\mathrm{mitochondrial}}}}\;{{{\mathrm{respiration}}}}} \right)\end{array}$$

$$\begin{array}{l}{{{\mathrm{ATP}}}}\;{{{\mathrm{production}} = ({\mathrm{last}}}}\;{{{\mathrm{rate}}}}\;{{{\mathrm{measurement}}}}\;{{{\mathrm{before}}}}\;{{{\mathrm{oligomycin}}}}\;{{{\mathrm{injection}}}})\\ \qquad \qquad\qquad\qquad\quad-\,\left( {{{{\mathrm{maximum}}}}\;{{{\mathrm{rate}}}}\;{{{\mathrm{measurement}}}}\;{{{\mathrm{after}}}}\;{{{\mathrm{oligomycin}}}}\;{{{\mathrm{injection}}}}} \right)\end{array}$$

For Seahorse XF Glycolysis Stress Test, ECAR was measured using the Seahorse XF Glycolysis Stress Test Kit (103020-100, Agilent Technologies, America) on an XFe96 Analyzer. Final concentrations of 10 mM glucose, 1 μM oligomycin, and 50 mM 2-deoxyglucose (2-DG) were used for all conditions. Glycolysis, glycolytic capacity, and glycolytic reserve were calculated as follows:

$$\begin{array}{l}{{{\mathrm{Glycolysis}} = ({\mathrm{maximum}}}}\;{{{\mathrm{rate}}}}\;{{{\mathrm{measurement}}}}\;{{{\mathrm{before}}}}\;{{{\mathrm{oligomycin}}}}\;{{{\mathrm{injection}}}})\\ \qquad\qquad\qquad- \,\left( {{{{\mathrm{last}}}}\;{{{\mathrm{rate}}}}\;{{{\mathrm{measurement}}}}\;{{{\mathrm{before}}}}\;{{{\mathrm{glucose}}}}\;{{{\mathrm{injection}}}}} \right)\end{array}$$

$$\begin{array}{l}{{{\mathrm{Glycolytic}}}}\;{{{\mathrm{capacity}}}} = \left( {{{{\mathrm{maximum}}}}\;{{{\mathrm{rate}}}}\;{{{\mathrm{measurement}}}}\;{{{\mathrm{after}}}}\;{{{\mathrm{oligomycin}}}}\;{{{\mathrm{injection}}}}} \right)\\ \qquad\qquad\qquad\qquad\qquad-\, \left( {{{{\mathrm{last}}}}\;{{{\mathrm{rate}}}}\;{{{\mathrm{measurement}}}}\;{{{\mathrm{before}}}}\;{{{\mathrm{glucose}}}}\;{{{\mathrm{injection}}}}} \right)\end{array}$$

$${{{\mathrm{Glycolytic}}}}\;{{{\mathrm{reserve}} = ({\mathrm{glycolytic}}}}\;{{{\mathrm{capacity}}}}) - \left( {{{{\mathrm{glycolysis}}}}} \right)$$

### Luciferase reporter assay

The *FA2H* promoter sequence was synthesized and cloned into pGL3-basic plasmid at Synbio Tech. The mutant *FA2H* promoter plasmids were constructed based on the binding motifs of FOXC2 from JASPAR database (http://jaspar.genereg.net/). Cells were seeded in 96-well plates, and transiently co-transfected with a mixture of *FA2H* promoter-Luc (wild or mutant type), pGL3-Luc, FOXC2 expressing vector, negative control vector (LV105), and Renilla luciferase (RL) reporter vectors (pRL-TK) as indicated. A site-directed mutagenesis kit (SBS, Shanghai, SDM-15) was used to generate mutant constructs for *FA2H* promoter. The Dual-Luciferase Reporter Assay System (Promega) was used to detect luciferase activities 48 hours after transfection. Details of luciferase reporter assays were performed according to the manufacturer’s protocol, and transfection efficiency was normalized by pRL-TK Renilla luciferase reporter.

### Statistical analysis

Data analysis was conducted using GraphPad Prism (Version 7). Statistical significance was determined by using two-sided Student’s *t*-test or Wilcoxon matched-pairs signed rank test. ANOVA or Friedman test was performed in multiple experimental groups. For the functional assays in vitro, experiments were independently performed at least three times. The correlation between the expression of FOXC2 and FA2H was determined by Spearman’s Rank Correlation test. For TCGA dataset analysis, the mRNA expression of indicated genes in normal esophageal mucosa tissues from the GTEx dataset was incorporated for statistical analysis. The data were reported as mean ± S.D. *P*-value <0.05 was considered statistically significant.

## Supplementary information

Supplementary_Information

Supplementary table 1

Supplementary table 2

Supplementary table 3

Supplementary table 4

The raw data of Western blot

## Data Availability

RNA-seq data have been deposited in the GEO database under the access code GSE182090. GEO reviewer token (wfsxgyigpdkfjyr) can be used for reviewers to review record GSE182090. The raw data of lipidomic profiling in FA2H-depleted cells and control cells were available from authors upon request. The ESCA data were derived from the TCGA Research Network (http://cancergenome.nih.gov/), which was further analyzed by UCSC Xena Browser (http://xena.ucsc.edu/) and GEPIA2 tool (http://gepia2.cancer-pku.cn/).
