# Correction: Dysregulated ceramides metabolism by fatty acid 2-hydroxylase exposes a metabolic vulnerability to target cancer metastasis

**DOI:** 10.1038/s41392-025-02379-5

**Published:** 2025-09-07

**Authors:** Xuantong Zhou, Furong Huang, Gang Ma, Wenqing Wei, Nan Wu, Zhihua Liu

**Affiliations:** 1 State Key Lab of Molecular Oncology, National Cancer Center/National Clinical Research Center for Cancer/Cancer Hospital, Chinese Academy of Medical Sciences and Peking Union Medical College, 100021 Beijing, China; 2 Key Laboratory of Carcinogenesis and Translational Research (Ministry of Education/Beijing), Department of Thoracic Surgery II, Peking University Cancer Hospital & Institute, 100142 Beijing, China; 3 Department of Gastric Surgery, Tianjin Medical University Cancer Institute and Hospital, National Clinical Research Center for Cancer; Key Laboratory of Cancer Prevention and Therapy, Tianjin; Tianjin’s Clinical Research Center for Cancer, 300060 Tianjin, PR China

**Keywords:** Drug development, Cancer metabolism, Metastasis, Gastrointestinal cancer, Oncogenes

Correction to: *Signal Transduction and Targeted Therapy* 10.1038/s41392-022-01199-1, published online 24 October 2022

Following online publication of the article,^1^ unintentional errors were identified in Fig. 6g, Supplementary Fig. S5f, and S10c. For Fig. 6g, upon careful examination of the raw data, the image panel representing the TNFα + Cer(d18:0/24:1) 100 μM treatment condition were mistakenly placed under the TNFα + Cer(d18:0/24:1) 50 μM treatment group in Fig. 6g, causing the inadvertent errors in the panel of K30P cells (TNFα + Cer(d18:0/24:1) 50 μM treatment) invasion in Fig. 6g, which is partially overlapped with the panel of the K30P cells (TNFα + Cer(d18:0/24:1) 100 μM treatment) invasion in Fig. 6g. Additionally, an inadvertent image placement error was identified in the panel of the K30P cells (TNFα + Cer(d18:0/24:1) 300 μM treatment) migration. The incorrect figures and corrected figures are provided as follows. The corrections have no impact on the original conclusions of the study. We apologize for this inadvertent mistake.

Incorrect Figure 6g
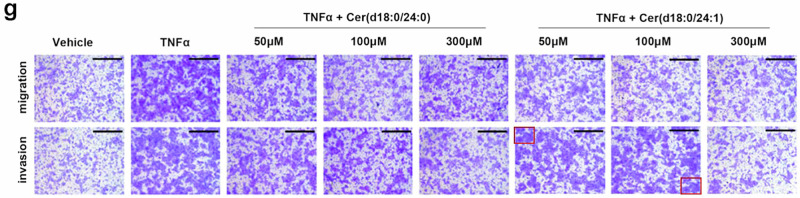


Correct Figure 6g
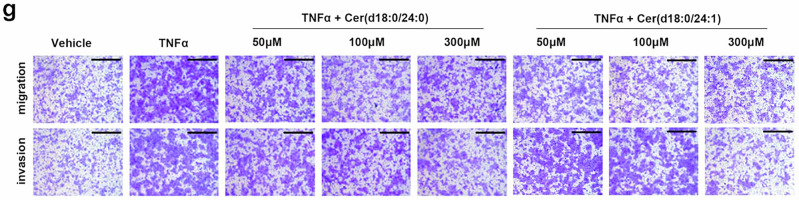


For Supplementary Fig. S5f, regarding that fenretinide at a concentration of 1 μM did not significantly inhibit the proliferation of K30LM3, as shown in Fig. S5e. In contrast, 100 μM ceramides and 2.5 μM fenretinide—concentrations used in subsequent functional assays—consistently suppressed both proliferation and migration in vitro, as demonstrated in Fig. S5b. Since fenretinide served as the positive control in our experiments, it is crucial that its effects reflect those of ceramide, particularly on attenuating both proliferation and metastasis. Considering the described results, we have removed the data for 1 μM and 5 μM Fenretinide in the transwell assay in the revised figure to avoid any potential misinterpretation. The incorrect figures and corrected figures are provided as follows. The corrections have no impact on the original conclusions of the study.

Incorrect section of Figure S5f
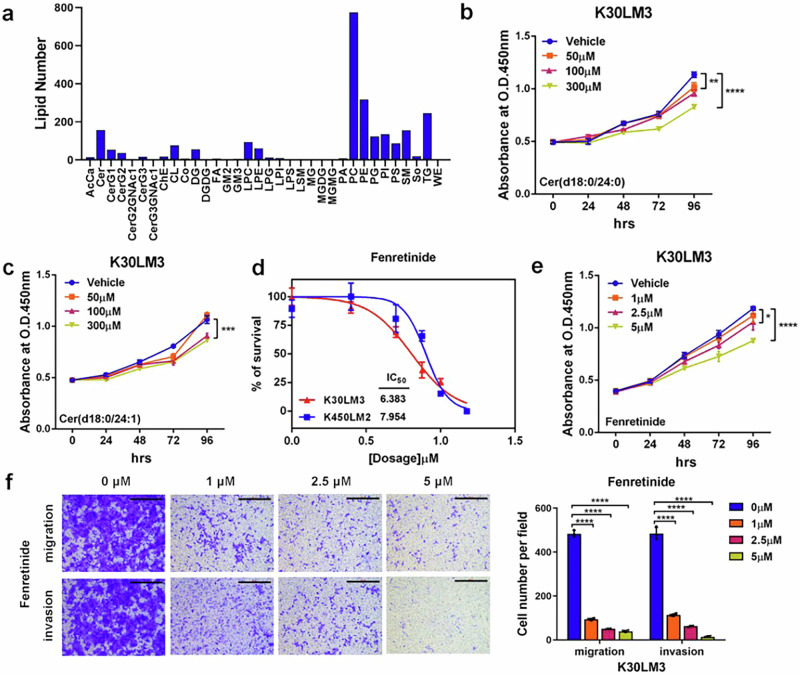


Correct Figure S5f
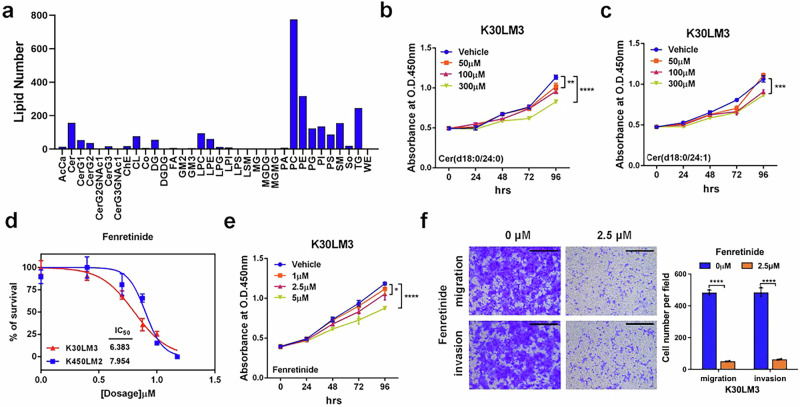


For Supplementary Fig. S10c, an inadvertent image placement error was identified. After examining the original image capture timestamps, we found that an error in the renaming process, combined with the computer's automatic naming functions, caused confusion, resulting in reversed and misplaced images in “TNFα” and “TNFα + Cer(d18:0/24:0) 50 μM” group, causing the inadvertent errors in the panel of K450P cells (TNFα treatment) migration in Fig. S9j, which is partially overlapped with the panel of the K450P cells (TNFα + Cer(d18:0/24:0) 50 μM treatment) migration in S10c. Since these represent analyses of the same cell samples from identical experimental groups, this does not constitute an error. Nevertheless, for the sake of rigor, we decided to re-present the K450P cells (TNFα treatment) migration in Fig. S10c. The incorrect figures and corrected figures are provided as follows. The corrections have no impact on the original conclusions of the study.

Incorrect section of Figure S10c and Figure S9j
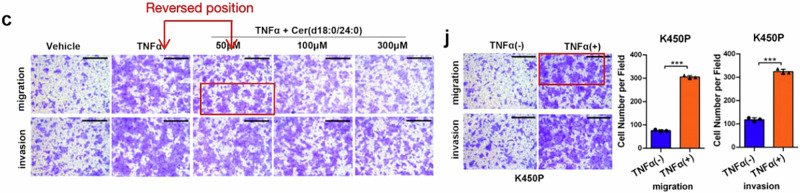


Correct Figure S10c
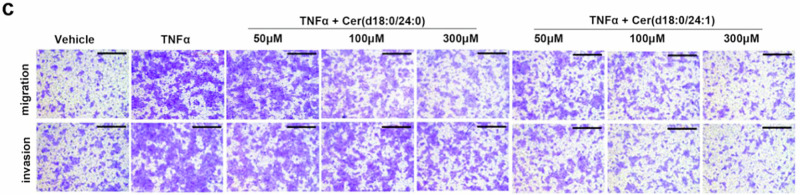


The original article has been corrected.

## Supplementary information

Incorrect section of Figure S5f

Correct Figure S5f

Incorrect section of Fig. S10c and Fig. S9j

Correct Figure S10c
