# Supplementary material for: Dysregulated ceramides metabolism by fatty acid 2-hydroxylase exposes a metabolic vulnerability to target cancer metastasis
Source: Signal Transduct Target Ther. 2022 Oct 24;7:370. doi: 10.1038/s41392-022-01199-1 (PMC9588768; doi:10.1038/s41392-022-01199-1)
Supplement: Supplementary file 1 — Supplementary_Information [file 41392_2022_1199_MOESM1_ESM.docx]

Supplementary Materials for

Dysregulated ceramides metabolism by fatty acid 2-hydroxylase exposes a metabolic vulnerability to target cancer metastasis

Xuantong Zhou^1,3^, Furong Huang^1^, Gang Ma^2^, Wenqing Wei^1^, Nan Wu^3✉^, Zhihua Liu^1✉­^

Correspondence to: Nan Wu (nanwu@bjmu.edu.cn), Zhihua Liu (liuzh@cicams.ac.cn)

**This PDF file includes:**

Materials and Methods

Figures. S1 to S10

References

Captions for Supplementary Tables 1-4

Caption for Data S1

Other Supplementary Materials for this manuscript include the following:

Supplementary Table 1. Information of primers

Supplementary Table 2. Differentially expressed genes identified from RNA-seq analysis

Supplementary Table 3. The results of Lipidomics

Supplementary Table 4. The raw data of figures as indicated

Data S1. The raw data of Western blot

Materials and Methods

Ethics statements

A commercial human ESCC tissue microarray was purchased from Outdo Biotech Company (#HEso-Squ150CS-02, Shanghai, China). Additionally, human ESCC tissue samples were obtained from patients receiving operation at Zhejiang Cancer Hospital (Hangzhou, China). All patients did not undergo neoadjuvant therapy. Informed consent was obtained from each enrolled patient. All human specimens were used in IHC experiments. This study was approved by the ethical committee of Chinese Academy of Medical Sciences Cancer Hospital (Beijing, China).

Five-week-old male SCID/Beige mice were purchased from Vital River Laboratories (Beijing, China). All mice used in this study were housed under specific pathogen-free (SPF) conditions. All procedures and experimental protocols were approved by the Institutional Animal Care and Use Committee of Chinese Academy of Medical Sciences Cancer Hospital (Beijing, China).

Cell cultured

The human ESCC cell lines KYSE30, KYSE450 were generously provided by Dr. Y. Shimada (Kyoto University, Kyoto, Japan) and maintained in RPMI 1640 supplemented with 10% fetal bovine serum (FBS) (HyClone, South Logan, UT, USA). HEK293T were purchased from the American Type Culture Collection (ATCC) (Manassas, VA) and maintained in DMEM supplemented with 10% fetal bovine serum (FBS) (HyClone, South Logan, UT, USA). For in vivo selection and bioluminescent imaging, KYSE30 and KYSE450 cells were infected with lentivirus expressing ﬁreﬂy luciferase and selected with G418 (200µg/mL) for two weeks.

Antibodies and reagents

Antibodies used in this study were as follows: Anti-FOXC2 (PA524588, Invitrogen), Anti-FA2H (15452-1-AP, Proteintech), Anti-GAPDH (2118, Cell Signaling Technology). Secondary antibodies included HRP Goat Anti-Mouse (926-80010, LI-COR) and HRP Goat Anti-Rabbit (926-80011, LI-COR). Cer(d18:0/24:0) and Cer(d18:0/24:1) were purchased from Avanti Lipids Polar. Fenretinide was purchased from MedChemExpress (HY-15373) and TNFα was purchased from Novus (#210-TA).

Animal study

All procedures and experimental protocols were approved by the Institutional Animal Care and Use Committee of Chinese Academy of Medical Sciences Cancer Hospital.

For the establishment of highly lung metastatic ESCC cell lines, *in vivo* selection was followed as previously described[^1^](#_ENREF_1). Briefly, 1x10^6^ K30P and K450P cells were intravenously injected into SCID/Beige mice. For the 1st round selection, lung metastatic lesions were observed after 3 months for both cells. Mice were then sacrificed and metastatic nodules were dissected and dissociated into pieces and grown in the culture dishes. G418 were used to select and differentiate tumor cells from fibroblast and other stromal cells. These G418-resistant tumor cells were denoted as first generation of lung metastatic derivatives (LM1). Similarly, LM1 cells were subjected to another round of in vivo selection by injecting them into the SCID/Beige mice, which gave rise to lung metastatic nodes and the second generation of lung metastatic derivatives (LM2). Accordingly, K30P or K450P were subjected to three or two round of *in vivo* selection, namely K30LM3 and K450LM2 respectively.

For experimental lung metastasis assay, 6-week-old male SCID/Beige mice were purchased from Vital River (Beijing, China). 1x10^6^ K30LM3 cells with stable FOXC2/FA2H depletion or FOXC2-depleted K30LM3 cells with additional FA2H overexpression and control cells were established and injected into the lateral tail veins of SCID/Beige mice. The pulmonary colonization of tumor cells were monitored by bioluminescent imaging after 40 days.

For dihydroceramides-based anti-metastasis therapy, 1x10^6^ K30LM3 cells were first intravenously injected. After one week, vehicle, Cer(d18:0/24:0) or Cer(d18:0/24:1) was administered three times a week by oral gavage with a dose of 10.26 mg/kg/day. Fenretinide (120mg/kg/day) were treated as positive control. Mice body weights in each group were measured during each treatment.

For bioluminescent imaging, mice were anesthetized and injected with 10 µL/g of D-luciferin (PerkinElmer) in DPBS intraperitoneally. After 15 mins, bioluminescence (BLI) was captured via Xenogen Optical in vivo Imaging System (IVIS; Xenogen). BLI was normalized to background value, which was deﬁned from a luciferin-injected mice without tumor cells[^1^](#_ENREF_1).

To evaluate the toxicity of Cer(d18:0/24:0), Cer(d18:0/24:1) and Fenretinide treatment, the mice organs including hearts, livers, and kidneys were harvested and paraffin-embedded followed by H&E staining. Histological examination were performed to analyze the treatment related morphological changes.

Plasmids and lentivirus packaging

FOXC2 and FA2H cDNA clones were subcloned into the pLVX-puro plasmid. shRNA oligos targeting FOXC2 or FA2H and a non-targeting oligo control were engineered into pSIH-puro plasmid. For pLVX-puro lentivirus production, the packaging plasmids Δ8.9, pLP2 was used. For pSIH-puro lentivirus production, the packaging plasmids vSVG, pLP1 and pLP2 were used. The indicated packaging plasmids and lentiviral vectors were co-transfected into HEK293T cells. After 48h transfection, the supernatant containing lentivirus particles was collected and stored in aliquots at -80 °C. For lentivirus infection, cells were first treated with polybrene (5 µg/mL) (TR-1003, Sigma), then infected with the indicated lentivirus. Stable cell populations were established by selecting with puromycin (2 μg/mL) (540222, Sigma) for 2 weeks.

Transwell assay

Parental KYSE30 (6x10^4^ per insert) or KYSE450 cells (2x10^5^ per insert) as well as LM cells with indicated treatment were suspended in FBS-free RPMI1640 and seeded into the upper chambers with or without pre-coated matrigel (BD, Franklin Lakes, NJ, USA). The bottom chambers were added with RPMI 1640 medium supplemented with 10% FBS. After 24 hr incubation, the migratory or invasive cells were methanol-fixed and stained with crystal violet. Cells in three randomly selected fields were photographed and statistically analyzed.

RNA extraction and qRT–PCR

Total RNA was extracted with TRIzol reagent (Thermo Fisher Scientific). The cDNAs were obtained using Quantscript RT kit (Tiangen, Beijing, China) according to the manufacture’s protocol. Real-time RT-PCR was performed by using SYBR Premix Ex TaqTM II (TaKaRa, Japan) on Step-one plus real-time PCR system (Applied Biosystems, Foster City, CA, USA), according to the manufacturer’s instructions. Primers used are listed in Supplementary Table 1.

RNA-seq and data analysis

RNA-seq analysis was performed as previously described[^2^](#_ENREF_2). For comparison of gene expression profiles between parental K30P, K450P cells and metastatic K30LM3, K450LM2 cells, cells were collected with three biological replicated in each group. Total RNA was extracted with Trizol reagents (Thermo Fisher Scientific). For library preparation, Poly(A)+ mRNA was isolated and enriched using NEBNext Poly(A) mRNA Magnetic Isolation Module. The mRNA was then recovered for library generation with NEBNext ® Ultra™ Directional RNA Library Prep Kit for Illumina (NEB, E7420S) following the manufacturer’s instructions. The cDNA libraries were sequenced at WuXiNextCODE. FastQC was used to examine the raw reads quality. Read alignment was conducted using STAR (v2.5.1b), R package edgeR (v3.8.5) was used to determine relative transcript abundances and differentially expressed genes (DEGs) between sample pair. The DEGs (Fold change>2-fold or 1.5, P<0.05) are listed in Supplementary Table 2.

Lipidomics analysis

Total lipids were extracted from 1x10^7^ FA2H-depleted K30LM3 cells and control cells with six biological replicates in each group[^3^](#_ENREF_3). Briefly, cell pellets were mixed well with 200µL ultra-pure water. Following addition of 240µL methanol to the mixture, samples were vortexed and added with MTBE and vortexed again. After placed at room temperature for 20min, samples were centrifuged at 8,000g for 15min at 4℃. The resulting lipid fraction was carefully collected and dried with nitrogen gas. Dried samples can be stored in -80℃. Before LC/MS, the samples were redissolved with isopropanol and vortexed then centrifuged at 8000g for 15min at 4℃. The supernatant was then subjected to UHPLC Nexera LC-30A for analysis. LipidSearch software version 4.1(Thermo Scientific™) was applied for peak recognition, peak alignment and lipid characterization, quantitative processing with the following parameters (5 ppm precursor tolerance, 5% product ion threshold and deletion of lipid molecules with a RSD >30%). LipidSearch data was further normalized by total peak area normalization and lipid molecules with the missing value >50% in each group was deleted. After the process of Pareto-scaling, SIMPCA-P 14.1 (Umetrics, Umea, Sweden) software was applied for multivariate statistical analysis, including principal component analysis (PCA), partial least squares discriminant analysis (PLS-DA), as well as orthogonal partial least squares discriminant analysis (OPLS-DA). Identification of differentially expressed lipid species was based on the VIP (Variable Importance for the Projection) values from OPLS-DA model. The lipids with threshold of VIP>1, P<0.05 in the univariate statistical analysis (Student’s t-test) were considered as statistically significant. The results of Lipidomics were listed in Supplementary table 3.

Cell proliferation assay

Cell proliferation was quantified by CCK-8 assays. K30P, K450P, 30LM3 and 450LM2 with indicated treatment were seeded into 96-well plates (2 × 10^4^ cells/mL; 100 µL/well). Cell Proliferation Reagent CCK-8 (#CK04, Dojindo Molecular Technologies, Japan) was used for measuring cell proliferation. After 1 h of incubation at 37℃, absorbance at 450 nm was measured using a microplate reader (BioTek).

Immunohistochemistry assay

Tissue microarrays were stained with anti-FOXC2 (PA524588, Invitrogen) and anti-FA2H (15452-1-AP, Proteintech) antibodies. The representative images of IHC staining were captured by Aperio ScanScope (Leica, Nussloch, Germany).

Western Blotting

Western blot was performed according to the standard protocol. Briefly, cells were harvested and lysed in RIPA buffer (1% NP-40, 0.1% sodium dodecyl sulfate (SDS), 50 mM Tris–HCl pH 7.4, 150 mM NaCl, 0.5% sodium deoxycholate, 1 mM (EDTA), 1 ×proteinase inhibitor cocktail (Roche)) for 30 min on ice. The proteins were resolved on 10% SDS-PAGE and transferred onto PVDF membranes (Millipore). The membranes were blocked with 5% milk powder solution, then incubated with specific antibodies at 4°C overnight. Following incubation with secondary antibodies, immunoblots were visualized using the ImageQuant LAS-4000 System (GE). Antibodies for western blotting are listed in ‘Antibodies and reagents’.

ChIP assay

ChIP assay was performed as previously described[^4^](#_ENREF_4). Briefly, indicated cells were crosslinked with 1% formaldehyde for 10 min at room temperature and cell pellets were collected and subjected to sonication. The sheared chromatin was then diluted followed by immune clearance for 1h at 4°C. Immunoprecipitation was performed by adding 4 µg of specific antibodies and incubated at 4°C overnight. Next, protein A-Sepharose beads were added and incubated for at least 1 h with rotation. The beads were then washed sequentially for 10 min each in TSE I, TSE II and buffer III and finally twice with TE buffer. Chromatin complexes were eluted with elution buffer (1% SDS, 0.1 M NaHCO3) and crosslinking was reversed at 65°C overnight. DNA fragments were purified with the QIAquick PCR purification kit (28104, Qiagen) and used for quantitative PCR reactions with Power SYBR Green PCR Master Mix reagent (Applied Biosystems). Primers used for ChIP are listed in Supplementary Table 1.

Seahorse Metabolic assay

For all assays, cells were pretreated with 4mg/mL BSA, 100μM Cer(d18:0/24:0), 100μM Cer(d18:0/24:1) and 2.5μM fenretinide for 24 hours and then 5 × 10^3^ treated K30LM3 or 6 × 10^3^ treated K450LM2 cells were seeded in XF96 cell culture plates incubated in a 5% CO_2_ incubator at 37℃ overnight.

For Seahorse XF Cell Mito Stress Test, OCR was measured using the Seahorse XF Cell Mito Stress Test Kit (103015-100, Agilent Technologies, America) on an XFe96 Analyzer. Final concentrations of 2.5 μM oligomycin, 2 μM Carbonyl cyanide-4 (trifluoromethoxy) phenylhydrazone (FCCP) and 0.5 μM Rotenone/antimycin A (Rot/AA) were used for all conditions. Basal respiration, maximal respiration and ATP production were calculated as follows:

Basal respiration = (last rate measurement before first injection) − (non-mitochondrial respiration rate)

Maximal respiration = (maximum rate measurement after FCCP injection) − (non-mitochondrial respiration)

ATP production = (last rate measurement before oligomycin injection) − (maximum rate measurement after oligomycin injection)

For Seahorse XF Glycolysis Stress Test, ECAR was measured using the Seahorse XF Glycolysis Stress Test Kit (103020-100, Agilent Technologies, America) on an XFe96 Analyzer. Final concentrations of 10 mM glucose, 1 μM oligomycin and 50 mM 2-deoxyglucose (2-DG) were used for all conditions. Glycolysis, glycolytic capacity and glycolytic reserve were calculated as follows:

Glycolysis = (maximum rate measurement before oligomycin injection) − (last rate measurement before glucose injection)

Glycolytic capacity = (maximum rate measurement after oligomycin injection) − (last rate measurement before glucose injection)

Glycolytic reserve = (glycolytic capacity) − (glycolysis)

Luciferase Reporter assay

The *FA2H* promoter sequence was synthesized and cloned into pGL3-basic plasmid at Synbio Tech. The mutant *FA2H* promoter plasmids were constructed based on the binding motifs of FOXC2 from JASPAR database (http://jaspar.genereg.net/). Cells were seeded in 96-well plates, and transiently co-transfected with a mixture of *FA2H* promoter-Luc (wild or mutant type), pGL3-Luc, FOXC2 expressing vector, negative control vector (LV105) and Renilla luciferase (RL) reporter vectors (pRL-TK) as indicated. A site-directed mutagenesis kit (SBS, Shanghai, SDM-15) was used to generate mutant constructs for *FA2H* promoter. The Dual-Luciferase Reporter Assay System (Promega) was used to detect luciferase activities 48 hours after transfection. Details of luciferase reporter assays were performed according to the manufacturer’s protocol, and transfection efficiency was normalized by pRL-TK Renilla luciferase reporter.

Statistical analysis

Data analysis was conducted using GraphPad Prism (Version 7). Statistical significance was determined by using two-sided Student’s t-test or Wilcoxon matched-pairs signed rank test. ANOVA or Friedman test was performed in multiple experimental groups. For the functional assays *in vitro*, experiments were independently performed at least three times. The correlation between the expression of FOXC2 and FA2H was determined by Spearman’s Rank Correlation test. For TCGA dataset analysis, the mRNA expression of indicated genes in normal esophageal mucosa tissues from the GTEx dataset were incorporated for statistical analysis. The data was reported as mean ± S.D. P-value <0.05 was considered statistically signiﬁcant.


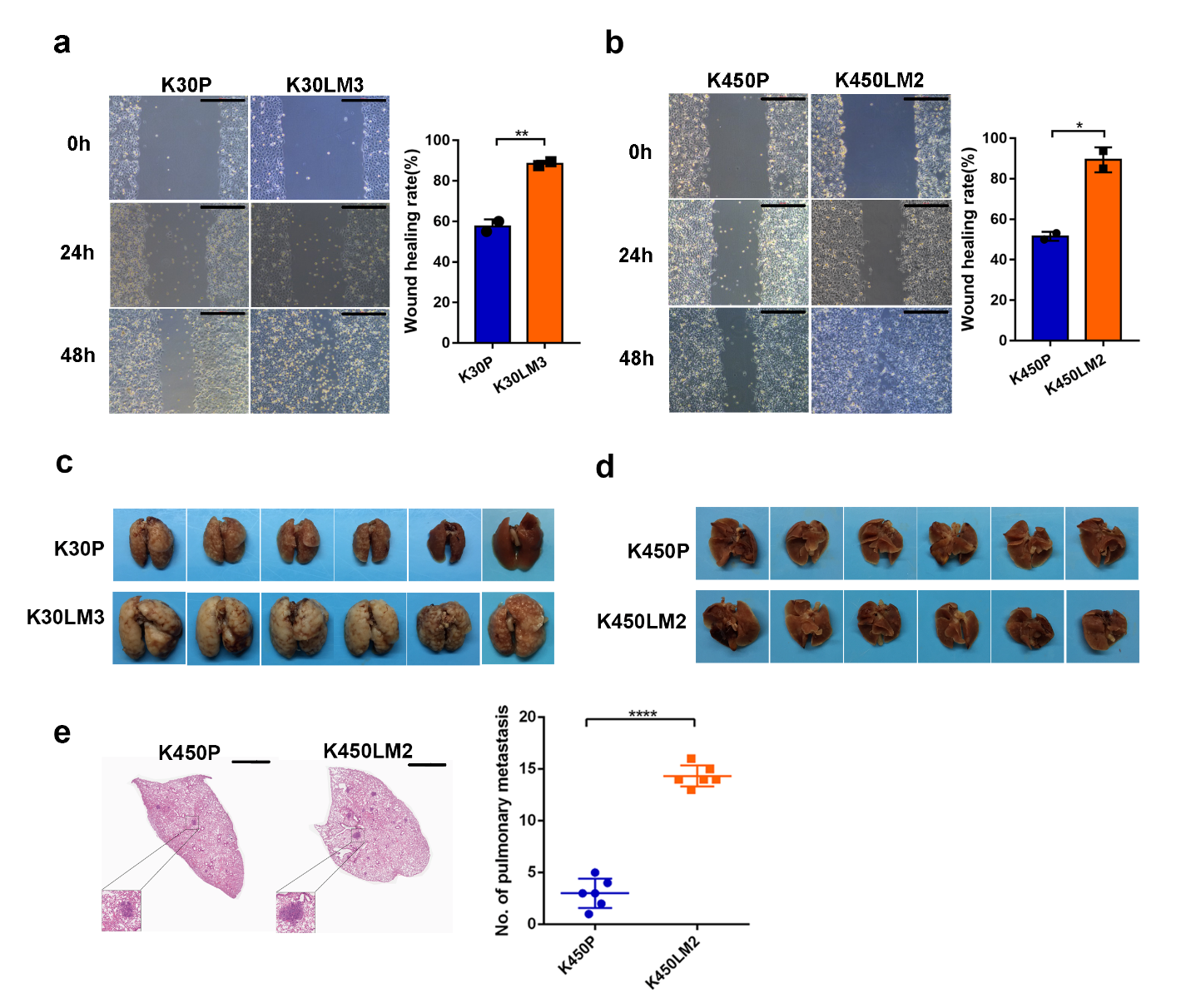


Figure. S1. Analysis of metastatic properties between LM cells and parental cells.

**a, b** Wound healing assay was performed to examine the migration ability of K30LM3/K30P cells (**a**) or K450LM2/K450P cells (**b**). Scale bar, 500µm. **c, d** Increased lung metastatic lesions in K30LM3 (**c**) or K450LM2 (**d**) injected mice in comparison with parental cells injected mice. **e** Representative H&E staining images show the lung metastatic lesions in K450P/K450LM2 injected mice (**left panel**). Scale bar, 2mm. Statistical analysis of lung weight between K450P and K450LM2 injected mice (**right panel**). Error bars denote mean ± SD. *P< 0.05, **P< 0.01, ****P< 0.0001.


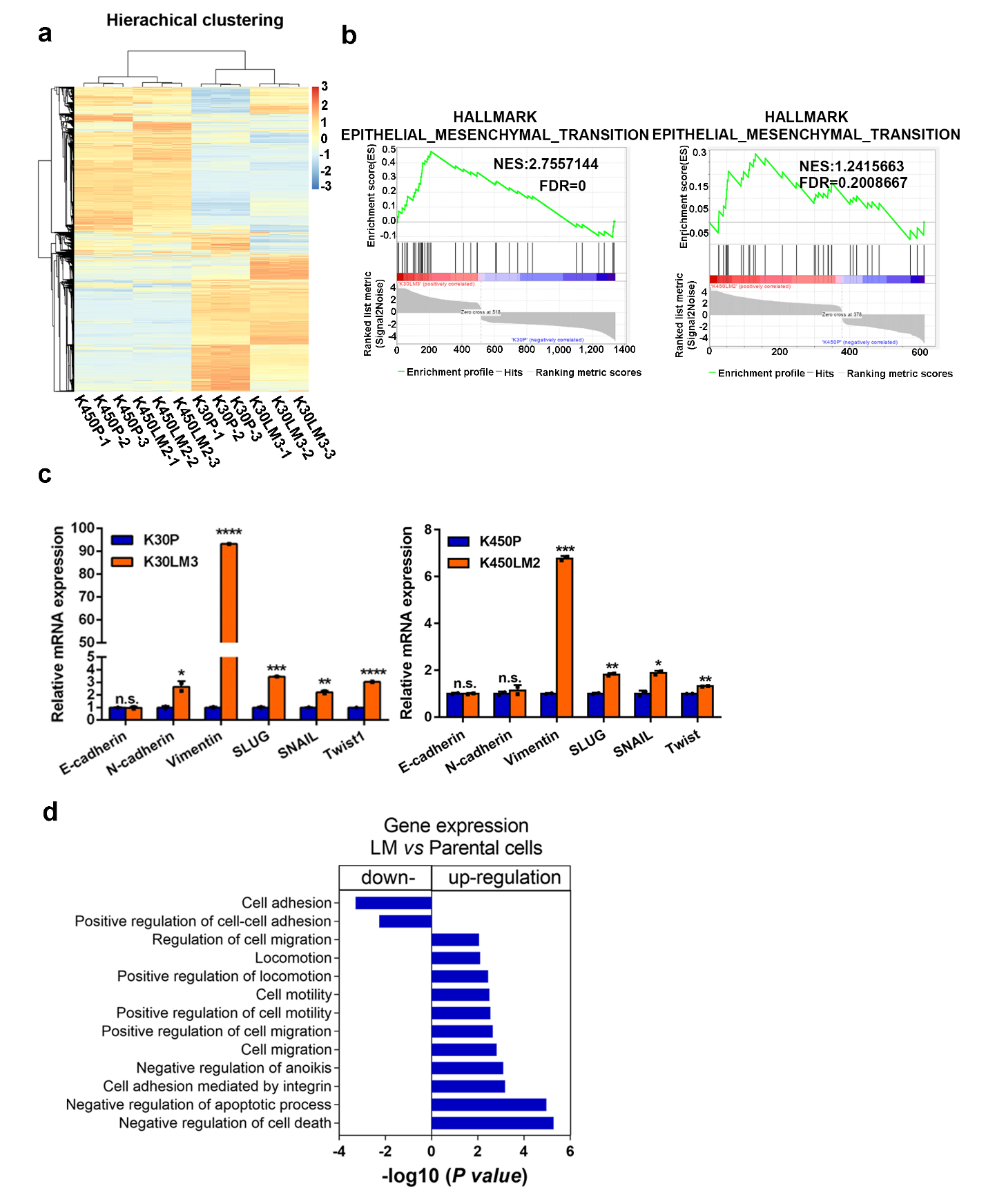


Figure. S2. Transcriptomic analysis between LM cells and parental cells.

**a** A heatmap of differentially expressed genes (FDR < 0.05, fold-change >2) between LM cells and parental cells. The scale bar is shown with the minimum expression value for each gene in blue and the maximum value in red. **b** Gene set enrichment analysis show epithelial mesenchymal transition (EMT) gene signature was significantly enriched in K30LM3 (**left panel**) or K450LM2 (**right panel**) cells relative to parental counterparts. Each of the black bars represents a gene in the pathway. **c** Quantitative RT-PCR analysis of key EMT signature genes expression between K30LM3 and K30P (**left panel**) or K450LM2 and K450P (**right panel**) cells. **d** Genomatix Biological Pathways analysis of co-regulated genes in LM cells relative to parental cells. Error bars denote mean ± SD. *P < 0.05, **P < 0.01, ***P < 0.001, ****P < 0.0001.


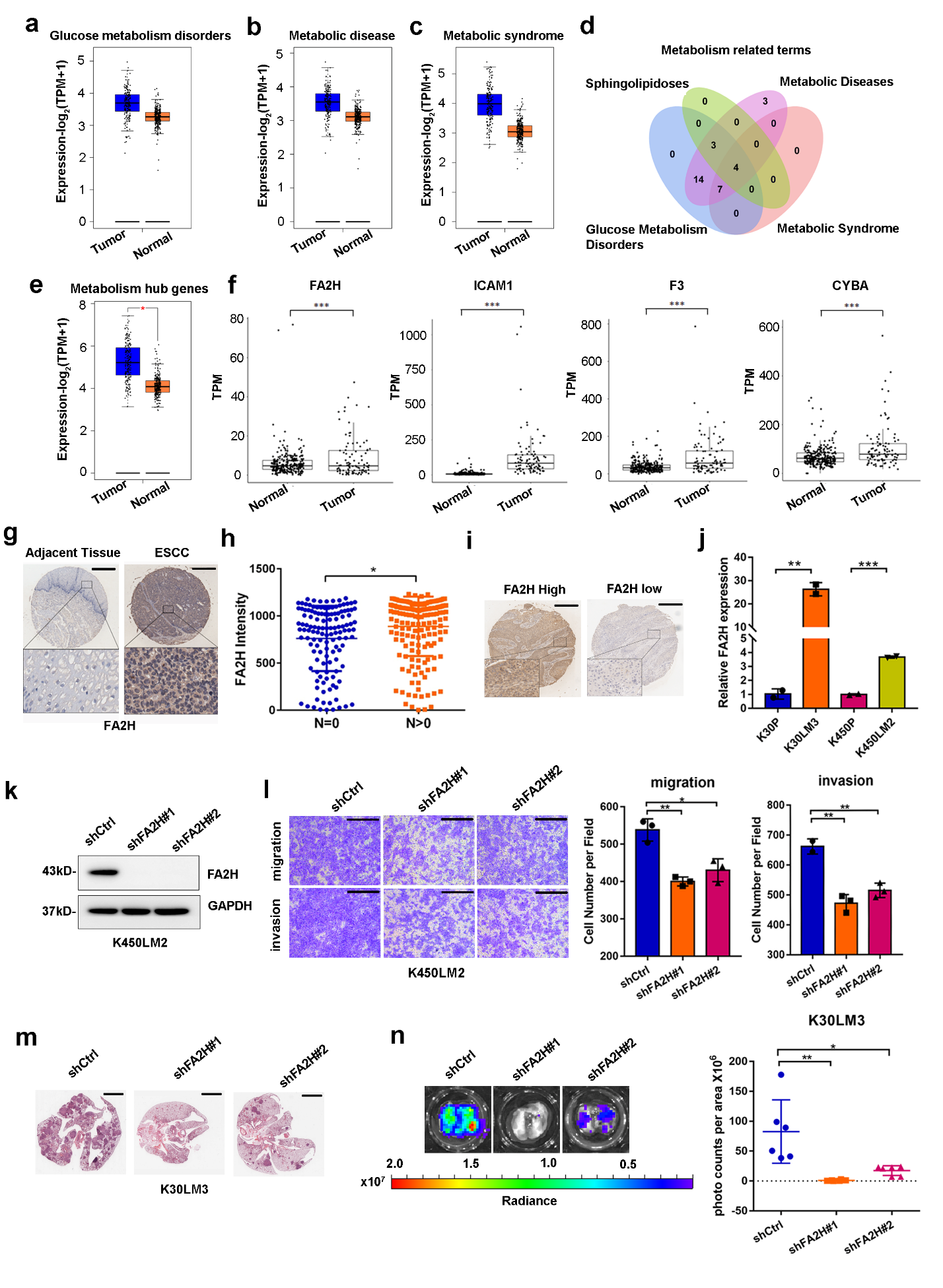


Figure. S3. Clinical significance and pro-metastatic role of FA2H in ESCC

**a-c** The gene signatures of glucose metabolism disorders (**a**), metabolic disease (**b**) and metabolic syndrome (**c**) were analyzed in TCGA ESCA datasets. **d** Co-regulated genes in overlapped metabolism-related pathways. **e** Metabolism hub genes (*FA2H , ICAM1, F3, CYBA*) signature is analyzed in the TCGA ESCA datasets. **f** The expressions of *FA2H, ICAM1, F3* and *CYBA* were analyzed in ESCC tissues from TCGA compared with matched normal tissues and esophageal mucosa tissues from GTEx dataset, respectively. **g** Representative images of H&E staining of FA2H protein in adjacent and ESCC tissues. Scale bar, 400µm. **h** Statistical analysis of FA2H staining intensity in ESCC tumors with or without lymph node metastasis (n = 306). **i** Representative images of FA2H staining intensity in ESCC tissues. Scale bar, 400µm. **j** Quantitative RT-PCR analysis of *FA2H* mRNA expression between LM cells and parental cells. **k** The FA2H knockdown efficiency was analyzed by western blotting in K450LM2 cells. **l** Transwell assay was performed to evaluate the effect of *FA2H* silencing on migration and invasion ability of K450LM2 cells. Scale bar, 500µm. **m** Representative H&E staining of lung sections corresponding to lung colonization by K30LM3 expressing shRNA targeting *FA2H* or a control hairpin (shCtrl). Scale bar, 3mm. **n** Bioluminescence quantification of lung tissues excised from tail-vain injected mice with K30LM3 expressing shRNA targeting *FA2H* or a control hairpin (shCtrl). Error bars denote mean ± SD. *P < 0.05, **P < 0.01, ***P < 0.001.


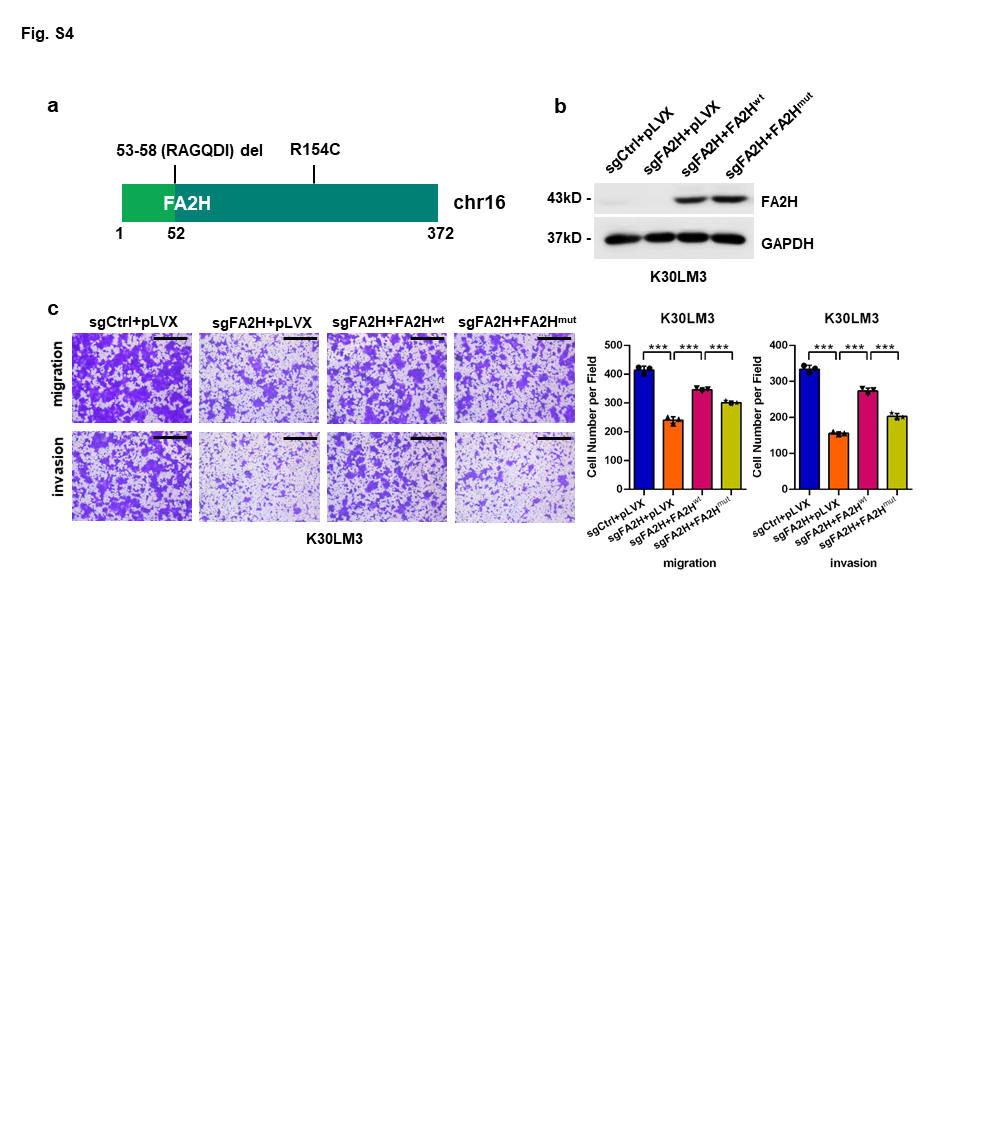


Figure. S4. The effect of FA2H^mut^ on the motility of K30LM3 cells.

**a** Schematic presentation of FA2H^mut^ construct. **b** Western blotting analysis of FA2H protein level in K30LM3 cells with treatment as indicated. **c** Transwell assay was performed to evaluate the effect of sgFA2H, FA2H^wt^ and FA2H^mut^ on migration and invasion ability of K30LM3 cells. Scale bar, 500µm. Error bars denote mean ± SD. ***P < 0.001.


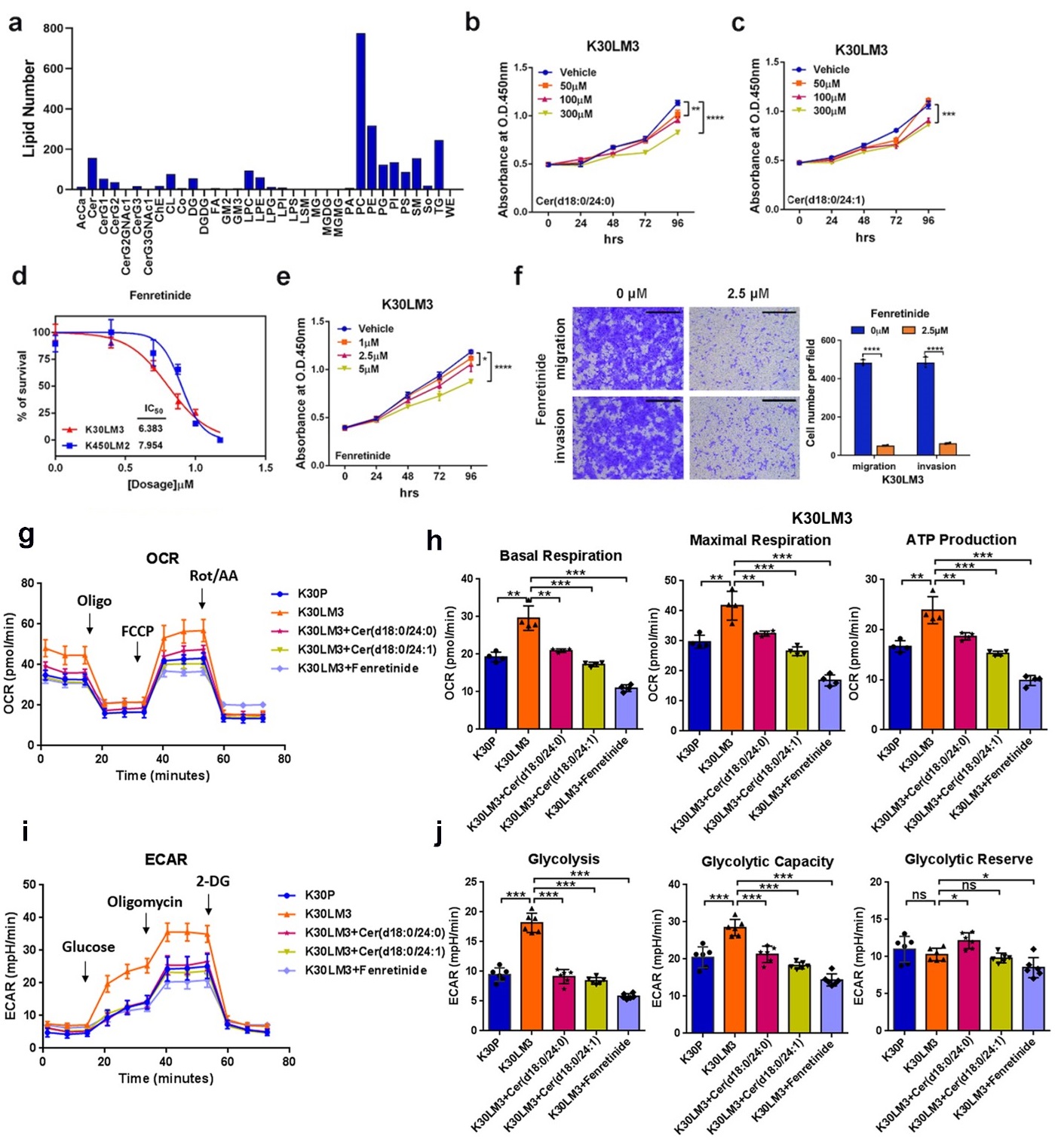


Figure. S5. The effect of Cer(d18:0/24:0), Cer(d18:0/24:1) or fenretinide treatment on the phenotypes of K30LM3 cells

**a** The lipid species identified from lipidomics of *FA2H*-depleted K30LM3 cells. **b, c** Measurement of K30LM3 cell proliferation under Cer(d18:0/24:0) (**b**) or Cer(d18:0/24:1) (**c**) treatment in a serial concentration by CCK-8 assay. **d** The IC_50_ value of fenretinide was measured in both K30LM3 and K450LM2 cells. **e** Measurement of K30LM3 cells proliferation under fenretinide treatment in a serial concentration by CCK-8 assay. **f** Transwell assay was performed to evaluate the effect of fenretinide on migration and invasion ability of K30LM3 cells. Scale bar, 500µm. **g** Oxygen consumption rate (OCR) of K30LM3 cells treated with Cer(d18:0/24:0), Cer(d18:0/24:1) or fenretinide were measured in real time under basal conditions and in response to the indicated inhibitors by seahorse. **h** Basal Respiration (**left panel**), Maximal Respiration (**middle panel**) and ATP production (**right panel**) of K30LM3 cells treated with Cer(d18:0/24:0), Cer(d18:0/24:1) or fenretinide were measured by mito stress test using XF seahorse analyzer. **i** Extracellular acidification rate (ECAR) of K30LM3 cells treated with Cer(d18:0/24:0), Cer(d18:0/24:1) or fenretinide were measured in real time under basal conditions and in response to the indicated inhibitors by seahorse. **j** Glycolysis (**left panel**), glycolytic capacity (**middle panel**) and glycolytic reserve (**right panel**) of K30LM3 cells treated with Cer (d18:0/24:0), Cer (d18:0/24:1) or fenretinide were measured by glycolysis stress test using XF seahorse analyzer. Error bars denote mean ± SD. *P < 0.05, **P < 0.01, ***P < 0.001, ****P< 0.0001.


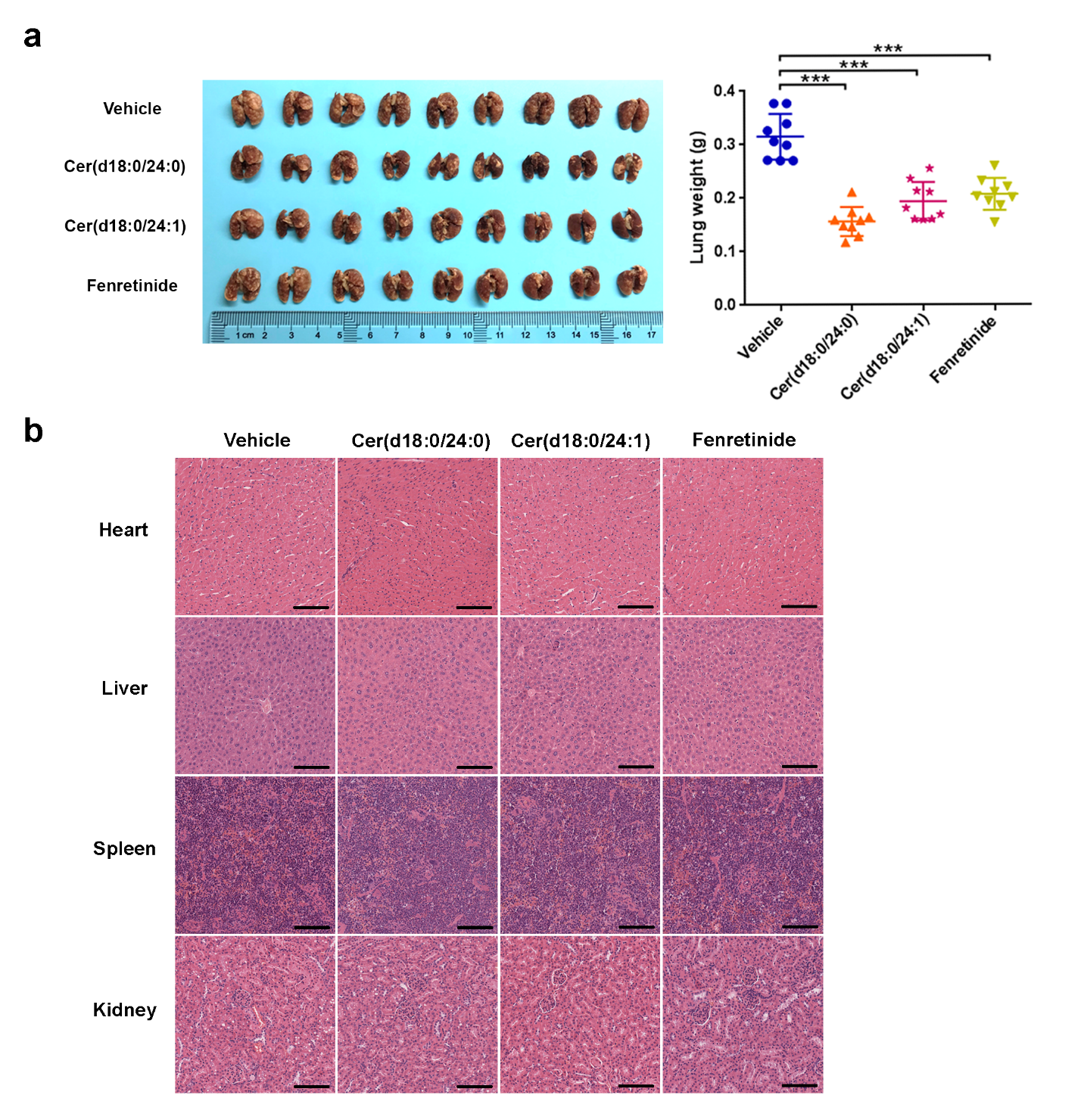


Figure. S6. Treatment of Cer(d18:0/24:0), Cer(d18:0/24:1) or fenretinide decreases the weights of lung metastases with no toxicity.

**a** Photographs of lungs harvested from mice tail-vain injected with K30LM3 cells followed by Cer(d18:0/24:0), Cer(d18:0/24:1), fenretinide or vehicle treatment in each group (**left panel**). Lung weight were then statistically analyzed (**right panel**). **b** Representative H&E images of vital organs harvested from mice tail-vain injected with K30LM3 cells followed by Cer(d18:0/24:0), Cer(d18:0/24:1), fenretinide or vehicle treatment in each group. Scale bar, 100µm. Error bars denote mean ± SD. ***P < 0.001.


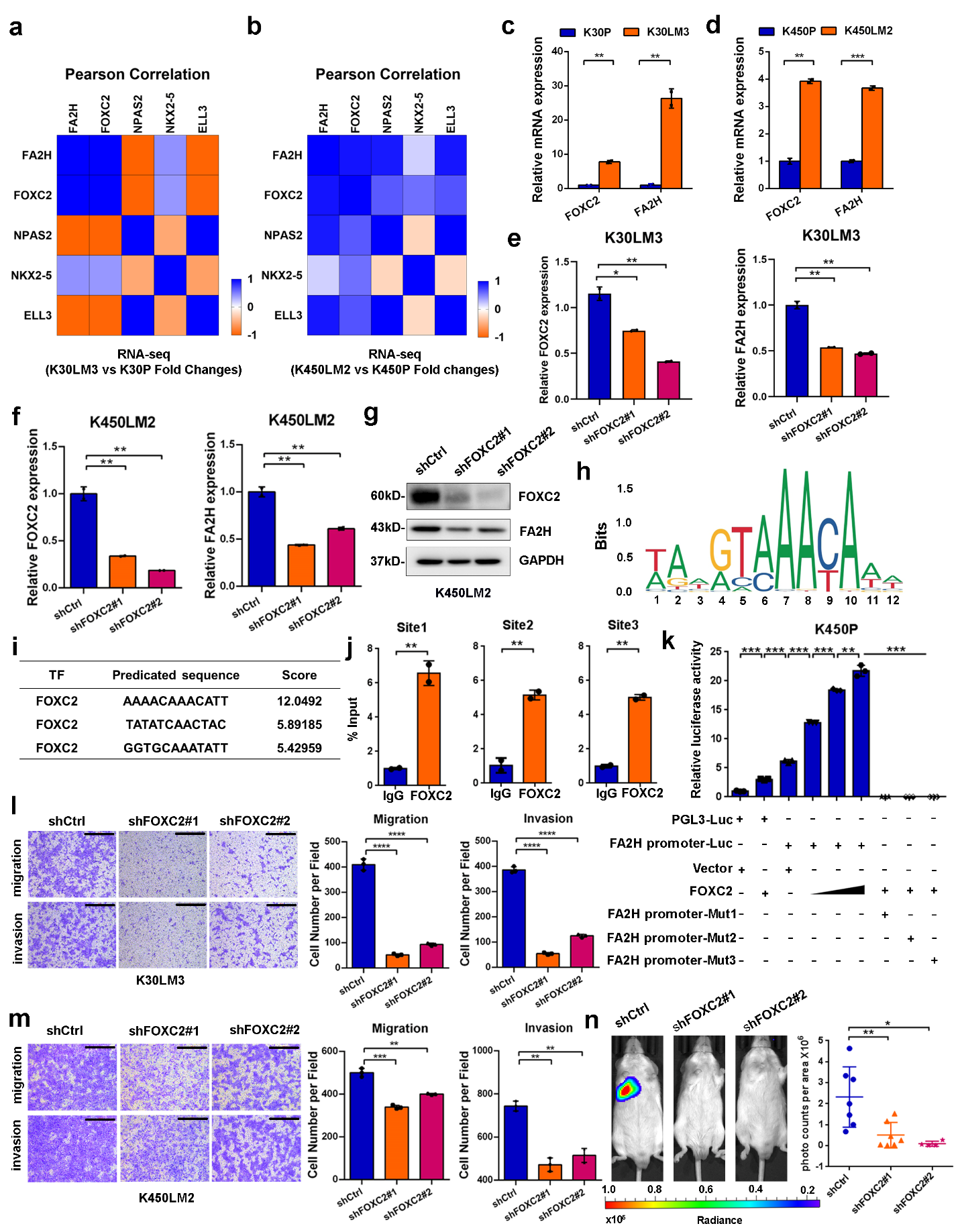


Figure. S7. FOXC2 transcriptionally upregulates *FA2H* and plays pro-metastatic roles in ESCC cells.

**a** Correlation analysis of co-regulated genes in RNA-seq data of K30LM3 and K30P cells. **b** Correlation analysis of co-regulated genes in RNA-seq data of K450LM2 and K450P cells. **c** Quantitative RT-PCR analysis of *FOXC2* and *FA2H* mRNA expression in K30LM3 and K30P cells. **d** Quantitative RT-PCR analysis of *FOXC2* and *FA2H* mRNA expression in K450LM2 and K450P cells. **e** Western blotting analysis of FOXC2 and FA2H protein level in FOXC2-depleted K450LM2 cells. **f** Quantitative RT-PCR analysis of *FOXC2* (**left panel**) and *FA2H* (**right panel**) mRNA expression in FOXC2-depleted K30LM3 cells. **g** Quantitative RT-PCR analysis of *FOXC2* (**left panel**) and *FA2H* (**right panel**) mRNA expression in FOXC2-depleted K450LM2 cells. **h** The binding motif recognized by FOXC2 was analyzed in JASPAR database. **i** The putative binding sequences of FOXC2 were analyzed in JASPAR database. **j** ChIP analysis of FOXC2 occupancy on the *FA2H* promoters in K450LM2 cells. **k** Luciferase activity of the pGL3-luciferase vector (PGL3-Luc), FA2H promoter-luciferase (FA2H promoter-Luc) or mutant FA2H promoter-luciferase (FA2H promoter-Mut1/2/3) reporter gene in K450P cells transiently transfected with FOXC2 or vector, n = 3 wells per group. **l** Transwell assay was performed to evaluate the effect of *FOXC2* silencing on migration and invasion ability of K30LM3 cells. Scale bar, 500µm. **m** Transwell assay was performed to evaluate the effect of *FOXC2* silencing on migration and invasion ability of K450LM2 cells. Scale bar, 500µm. **n** Bioluminescence quantification of lung colonization by K30LM3 expressing shRNA targeting *FOXC2*, or a control hairpin (shCtrl). Error bars denote mean ± SD. *P < 0.05, **P < 0.01, ***P < 0.001, ****P< 0.0001.


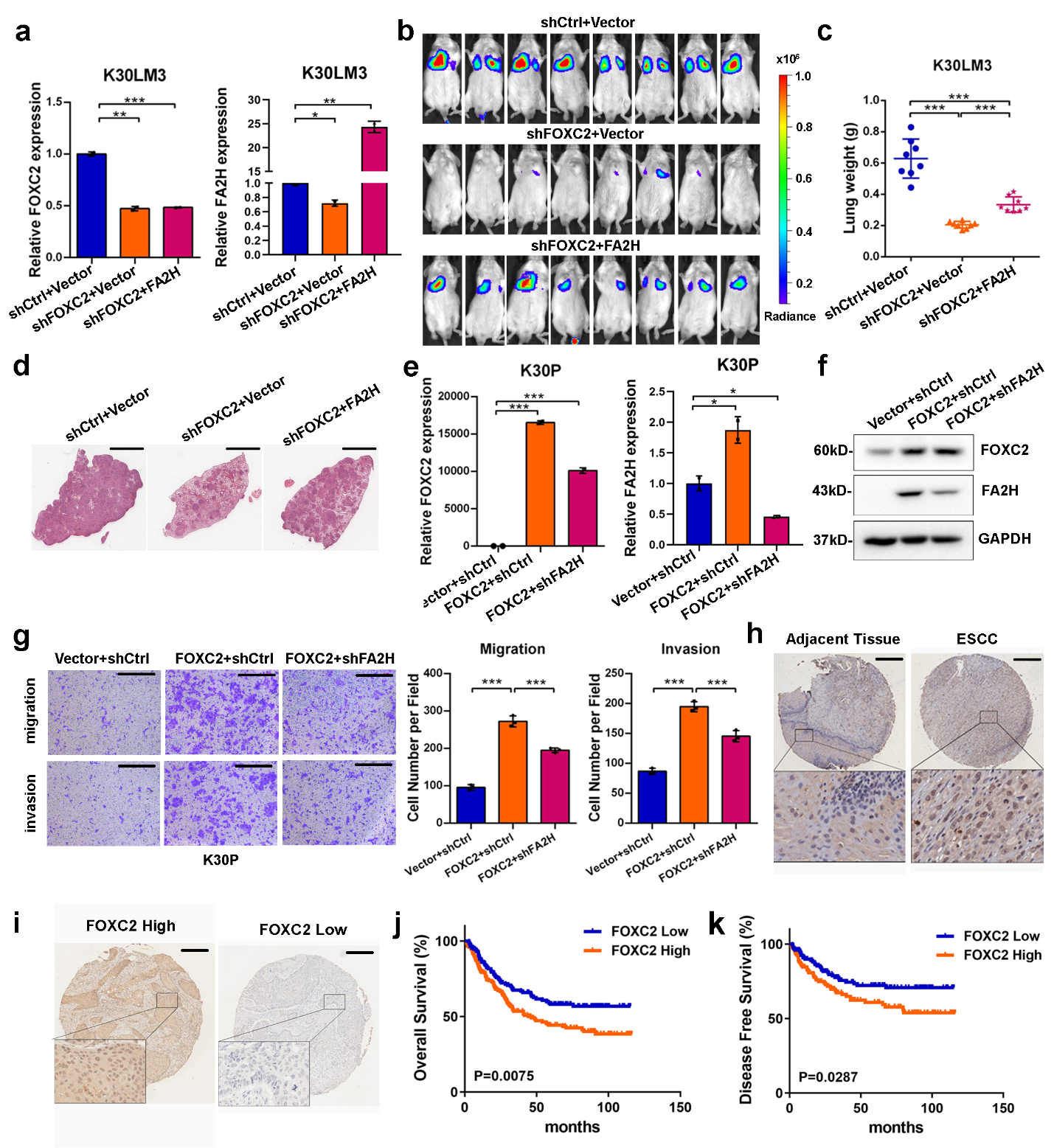


Figure. S8. Functional interplay between FOXC2 and FA2H in ESCC metastasis.

**a** Quantitative RT-PCR analysis of *FA2H* mRNA expression in K30LM3 expressing shRNA targeting *FOXC2*, shRNA targeting *FOXC2* with additional *FA2H* overexpression, or a control hairpin (shCtrl). **b** Bioluminescence imaging of mice intravenously injected with K30LM3 expressing shRNA targeting *FOXC2*, shRNA targeting *FOXC2* with additional *FA2H* overexpression, or a control hairpin (shCtrl). n = 8 mice per group. **c** Statistical analysis of lung weight from mice intravenously injected with K30LM3 expressing shRNA targeting *FOXC2*, shRNA targeting *FOXC2* with additional *FA2H* overexpression, or a control hairpin (shCtrl). **d** Representative H&E staining shows the lung metastatic lesions of mice among each group. Scale bar, 3mm. **e** Quantitative RT-PCR analysis of *FOXC2* and *FA2H* mRNA expression in K30P stably overexpressing *FOXC2* with or without additional *FA2H* depletion. **f** Western blotting analysis of FOXC2 and FA2H protein level in K30P stably overexpressing *FOXC2* with or without additional *FA2H* depletion. **g** Transwell assay was performed to evaluate the effect of *FOXC2* overexpression with or without additional *FA2H* depletion in K30P cells. Scale bar, 500µm. **h** Representative images of immunohistochemical staining of FOXC2 protein in adjacent and ESCC tissues. Scale bar, 400µm. **i** Representative images of FOXC2 staining intensity in ESCC tissues. Scale bar, 400µm. **j, k** Kaplan-Meier analysis of overall survival (**j**) and disease-free survival (**k**) in ESCC patients with high or low FOXC2 expression (n = 306). The high or low expression of FA2H or FOXC2 in Kaplan-Meier analysis was stratified by median FA2H or FOXC2 protein levels from IHC staining quantifications. Error bars denote mean ± SD. *P < 0.05, **P < 0.01, ***P < 0.001.


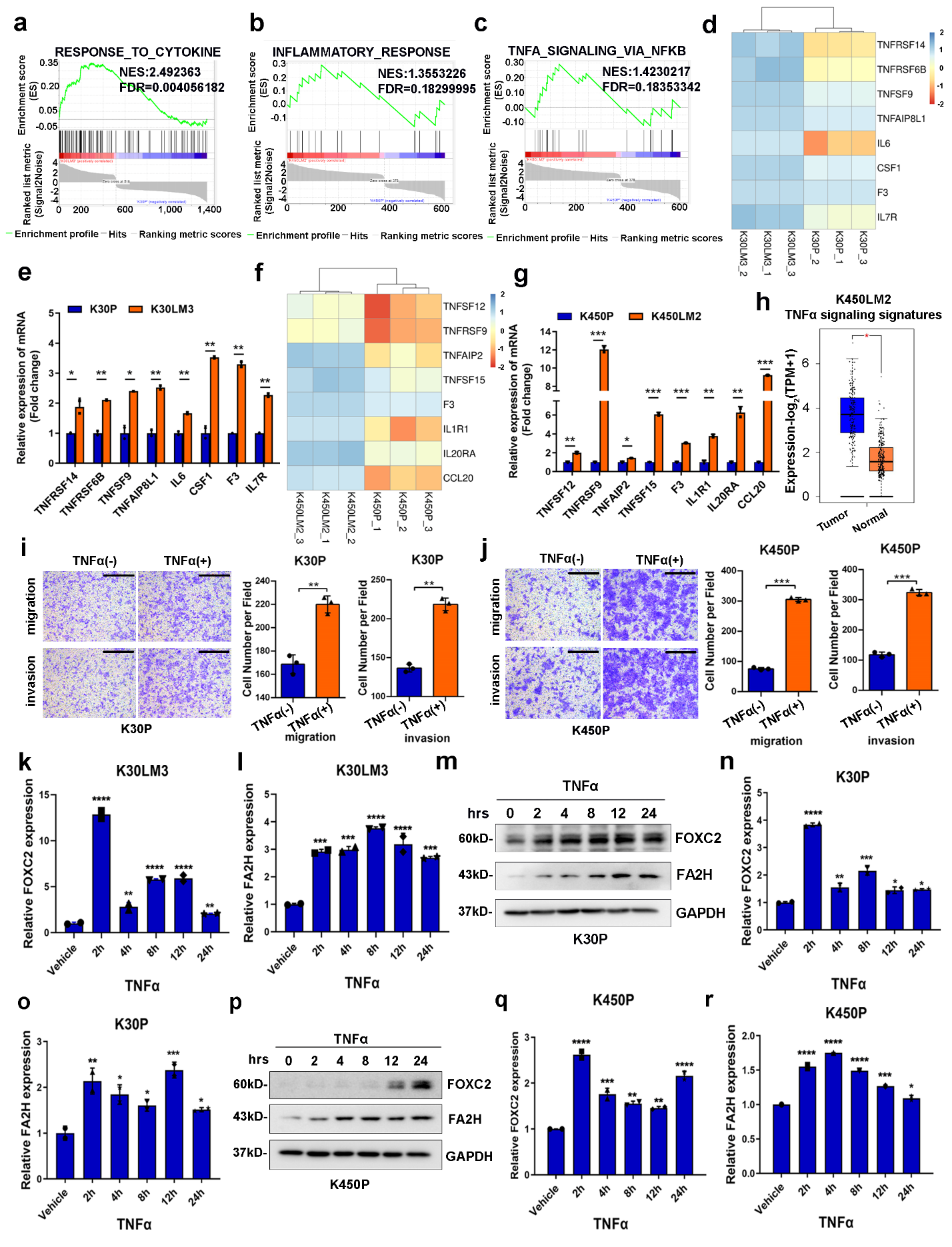


Figure. S9. TNFα is implicated in the regulation of FOXC2-FA2H axis

**a** Gene set enrichment analysis shows ‘response to cytokine’ was significantly enriched in K30LM3 relative to K30P cells. Each of the black bars represents a gene in the pathway. **b, c** Gene set enrichment analysis shows ‘inflammatory response’ (**b**) and ‘TNFα signaling via NF-κB’ (**c**) signatures were significantly enriched in K450LM2 relative to K450P cells. Each of the black bars represents a gene in the pathway. **d, e** A heatmap showing the differential expression of selected genes involved in ‘TNFα signaling via NF-κB’ and ‘response to cytokine’ pathway between K30LM3 and K30P cells (**d**), which were further validated by quantitative RT-PCR analysis (**e**). **f, g** A heatmap showing the differential expression of selected genes involved in ‘inflammatory response’ and ‘TNFα signaling via NF-κB’ between K450LM2 and K450P cells (**f**), which were further validated by quantitative RT-PCR analysis (**g**). **h** Analysis of TNFα signaling gene signature identified from K450LM2 cells in TCGA ESCA dataset using GEPIA2 tool. **i, j** Transwell assay was performed to evaluate the effect of TNFα on migration and invasion ability of K30P cells (**i**) and K450P cells (**j**). Scale bar, 500µm. **k, l** Quantitative RT-PCR analysis of *FOXC2* (**k**) and *FA2H* (**l**) mRNA expression in K30LM3 cells treated with TNFα at different time points.  **m** Western blotting analysis of FOXC2 and FA2H protein level after TNFα treatment at different time points in K30P cells. **n, o** Quantitative RT-PCR analysis of *FOXC2* (**n**) and *FA2H* (**o**) mRNA expression in K30P cells treated with TNFα at different time points. **p** Western blotting analysis of FOXC2 and FA2H protein level after TNFα treatment at different time points in K450P cells. **q, r** Quantitative RT-PCR analysis of *FOXC2* (**q**) and *FA2H* (**r**) mRNA expression in K450P cells treated with TNFα at different time points. Error bars denote mean ± SD. *P < 0.05, **P < 0.01, ***P < 0.001, ****P < 0.0001.


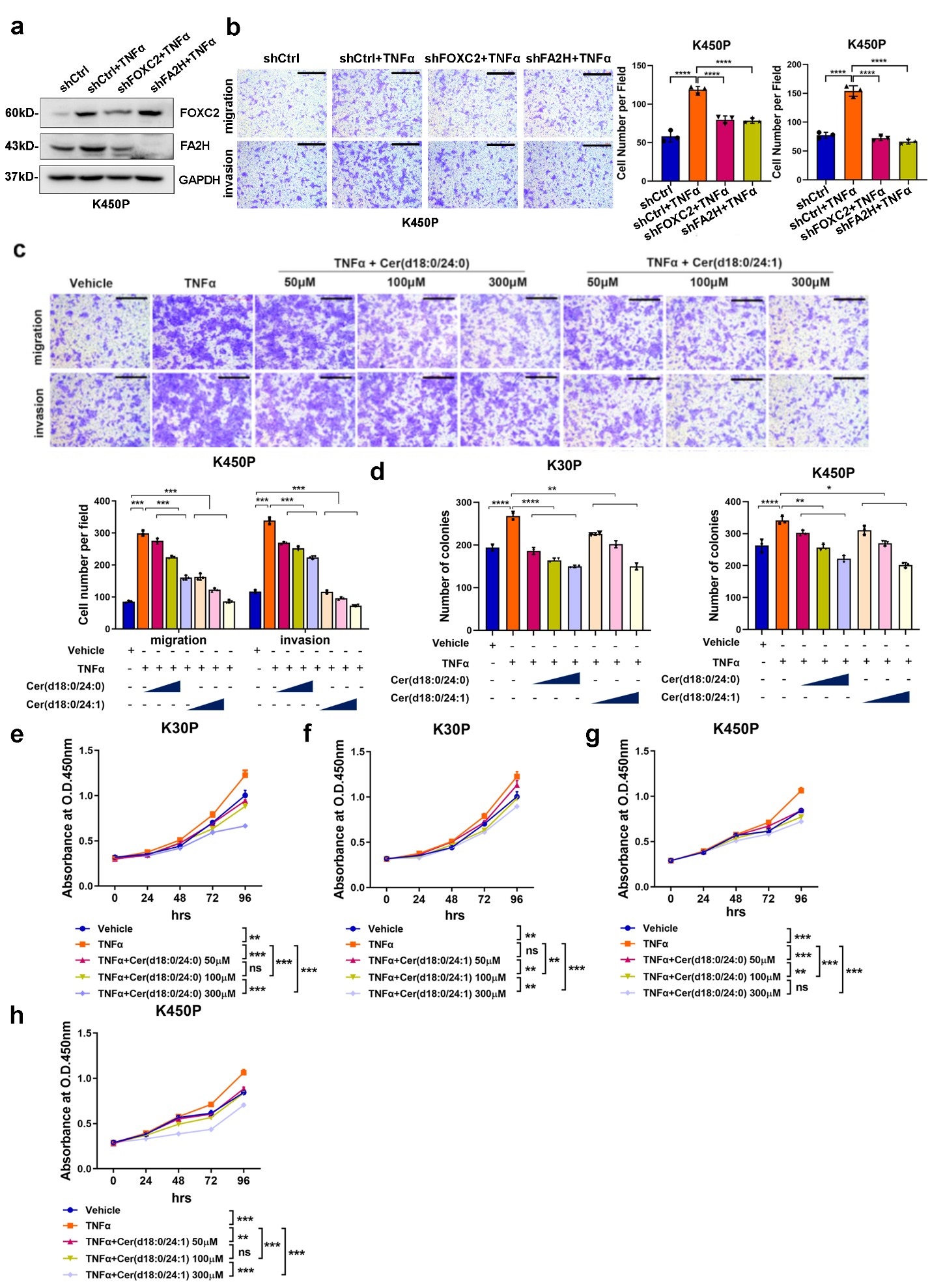


Figure. S10. Oncogenic functions of TNFα can be inhibited by depleting FOXC2-FA2H axis or treatment of Cer(d18:0/24:0) or Cer(d18:0/24:1).

**a** Western blotting analysis of TNFα-induced FOXC2 and FA2H protein level in K450P expressing shRNA targeting *FOXC2, FA2H* or a control hairpin (**shCtrl**). **b** Transwell assay was performed to evaluate the effect of TNFα-induced FOXC2 and FA2H expression on migration and invasion ability of K450P cells. Scale bar, 500µm. **c** Representative images of K450P transwell assay with TNFα treatment alone or in combination with Cer(d18:0/24:0) or Cer(d18:0/24:1) in a serial concentration. Scale bar, 500µm. **d** Measurement of K30P (**left panel**) and K450P (**right panel**) cell clonogenic ability under TNFα treatment alone or in combination with Cer(d18:0/24:0) or Cer(d18:0/24:1) in a serial concentration. **e, f** The K30P cell proliferation under TNFα treatment alone or in combination with Cer(d18:0/24:0) (**e**) or Cer(d18:0/24:1) (**f**) in a serial concentration was assessed by CCK-8 assay. **g, h** The K450P cell proliferation under TNFα treatment alone or in combination with Cer(d18:0/24:0) (**g**) or Cer(d18:0/24:1) (**h**) in a serial concentration was assessed by CCK-8 assay. Error bars denote mean ± SD. *P < 0.05, **P < 0.01, ***P < 0.001, ****P < 0.0001.

**References**

1 Minn, A. J. *et al.* Genes that mediate breast cancer metastasis to lung. *Nature* **436**, 518-524 (2005).

2 Li, X. *et al.* A S100A14-CCL2/CXCL5 signaling axis drives breast cancer metastasis. *Theranostics* **10**, 5687-5703 (2020).

3 Cantor, J. R. *et al.* Physiologic Medium Rewires Cellular Metabolism and Reveals Uric Acid as an Endogenous Inhibitor of UMP Synthase. *Cell* **169**, 258-272 e217 (2017).

4 Wang, Q. *et al.* A hierarchical network of transcription factors governs androgen receptor-dependent prostate cancer growth. *Mol. Cell* **27**, 380-392 (2007).

**Supplementary Table 1.**

Information of primers

**Supplementary Table 2.**

Differentially expressed genes identified from RNA-seq analysis

**Supplementary Table 3.**

The results of Lipidomics

**Supplementary Table 4.**

The raw data of figures as indicated

**Data S1.**

The raw data of Western blot
