# Supplementary material for: Dysregulated ceramides metabolism by fatty acid 2-hydroxylase exposes a metabolic vulnerability to target cancer metastasis
Source: Signal Transduct Target Ther. 2022 Oct 24;7:370. doi: 10.1038/s41392-022-01199-1 (PMC9588768; doi:10.1038/s41392-022-01199-1)
Supplement: Supplementary file 6 — The raw data of Western blot [file 41392_2022_1199_MOESM6_ESM.pptx]

## Slide 1
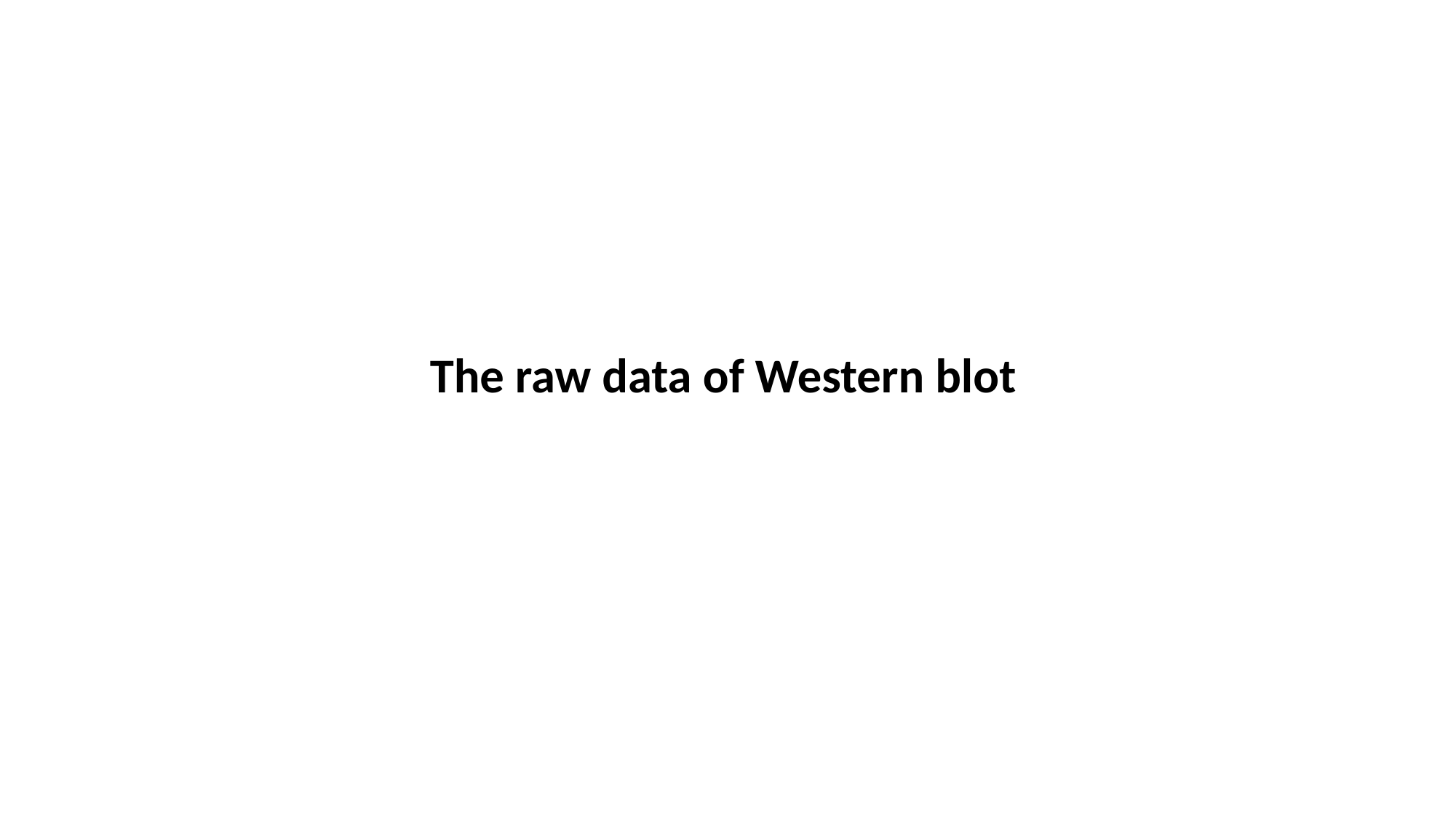

The raw data of Western blot

## Slide 2
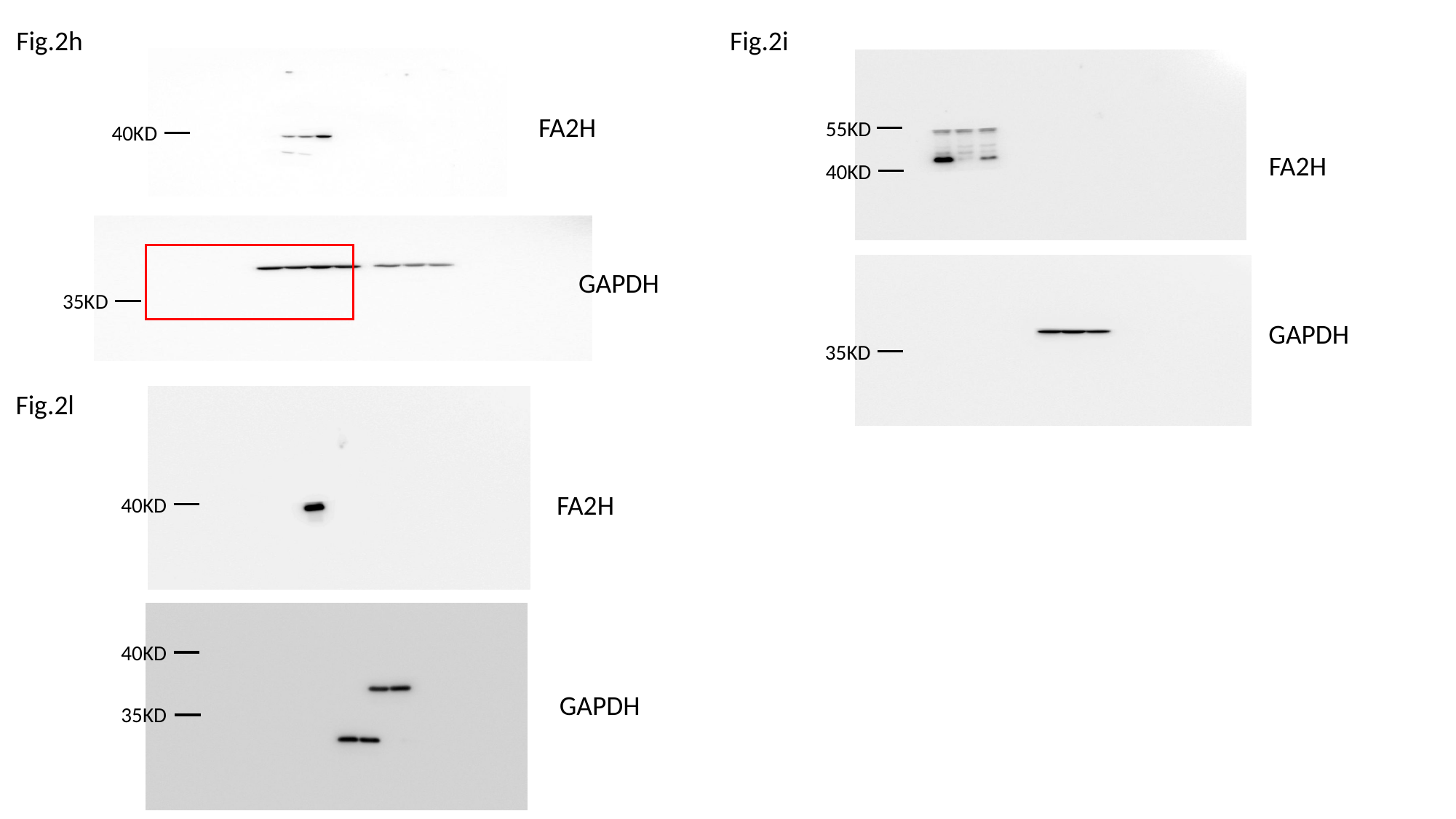

Fig.2h
Fig.2i
FA2H
40KD
GAPDH
35KD
55KD
FA2H
40KD
GAPDH
35KD
Fig.2l
40KD
35KD
FA2H
GAPDH
40KD

## Slide 3
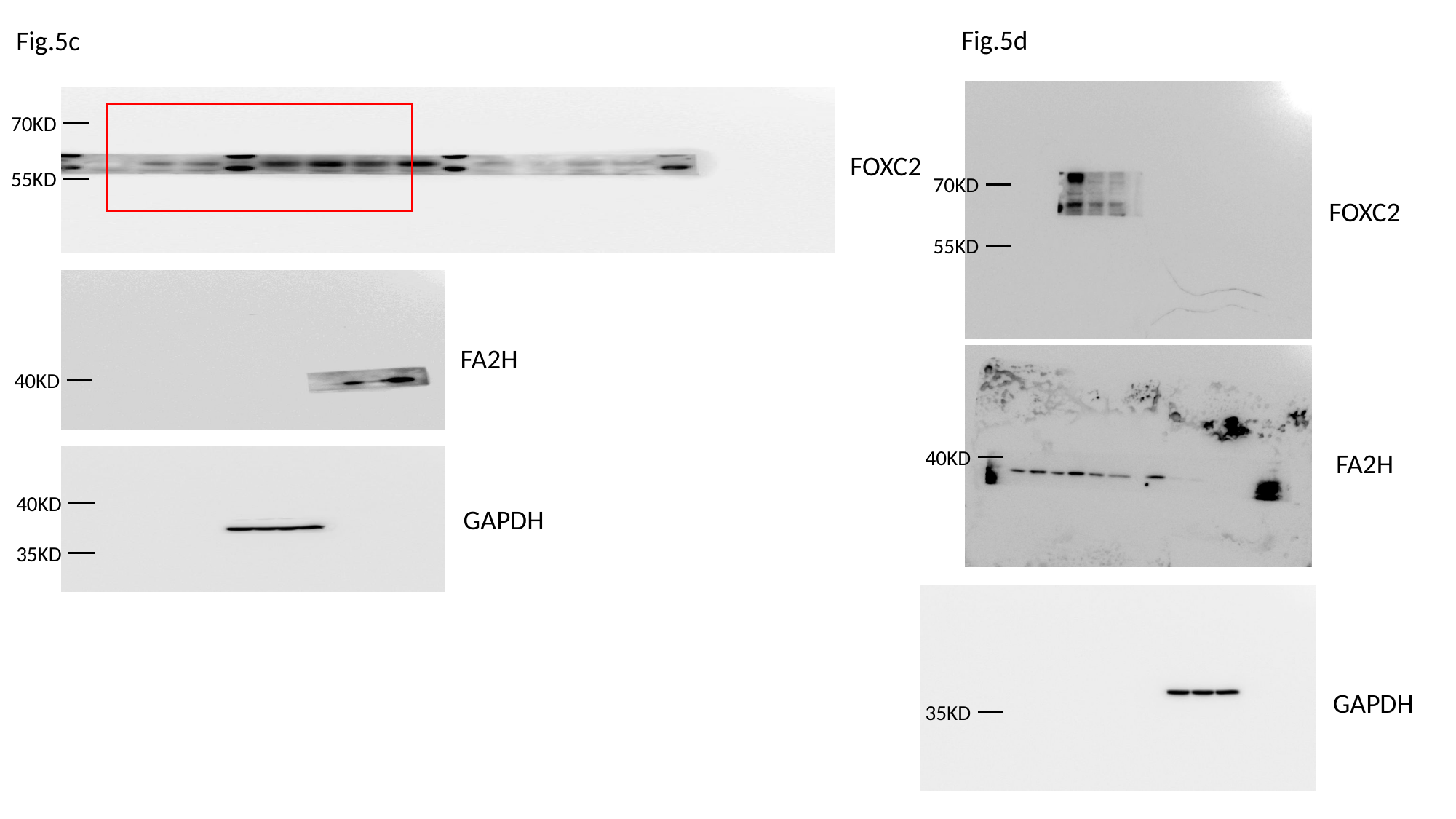

Fig.5d
Fig.5c
70KD
FOXC2
55KD
70KD
55KD
FOXC2
FA2H
40KD
40KD
GAPDH
35KD
40KD
FA2H
GAPDH
35KD

## Slide 4
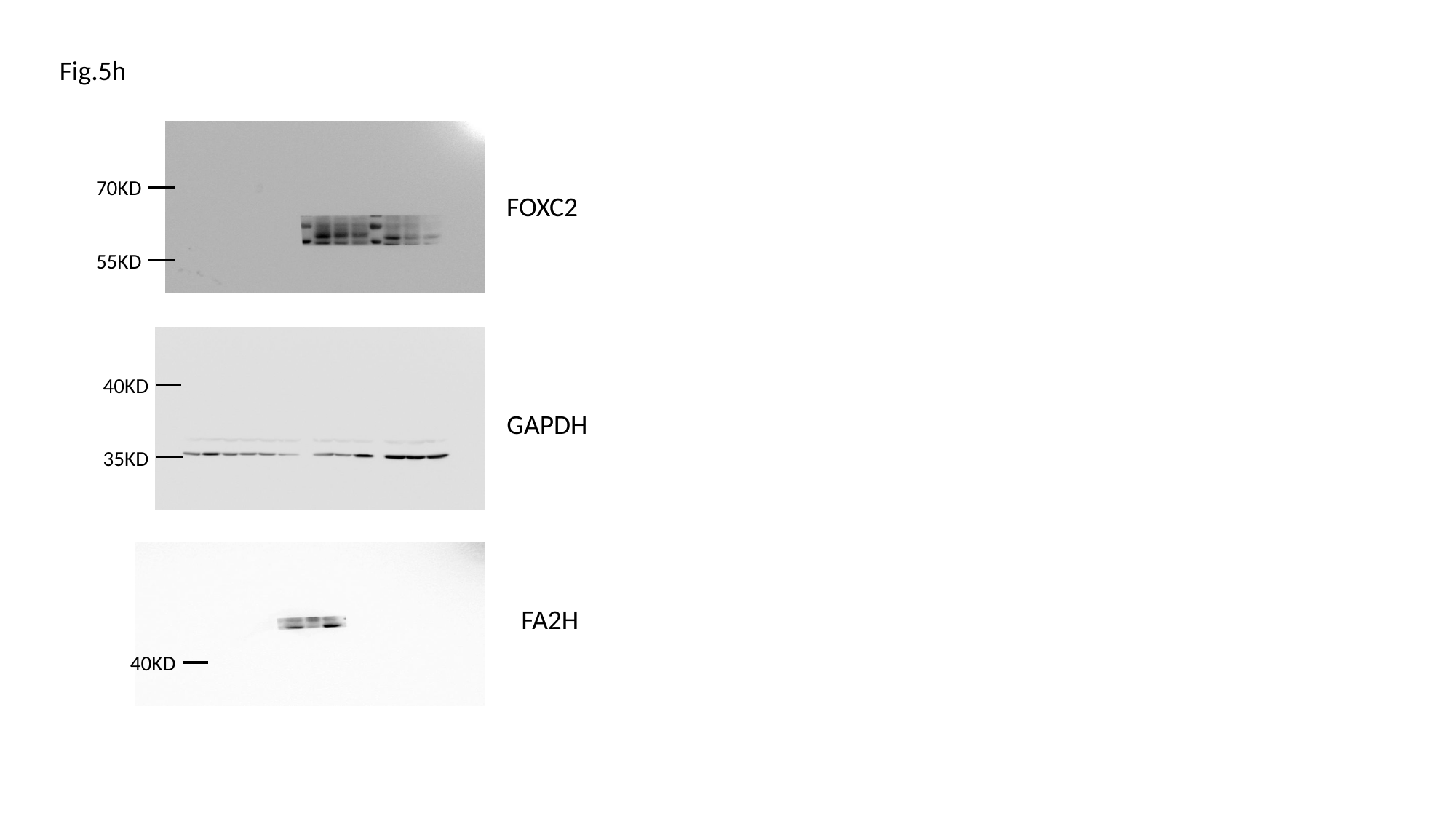

Fig.5h
70KD
FOXC2
55KD
40KD
GAPDH
35KD
FA2H
40KD

## Slide 5
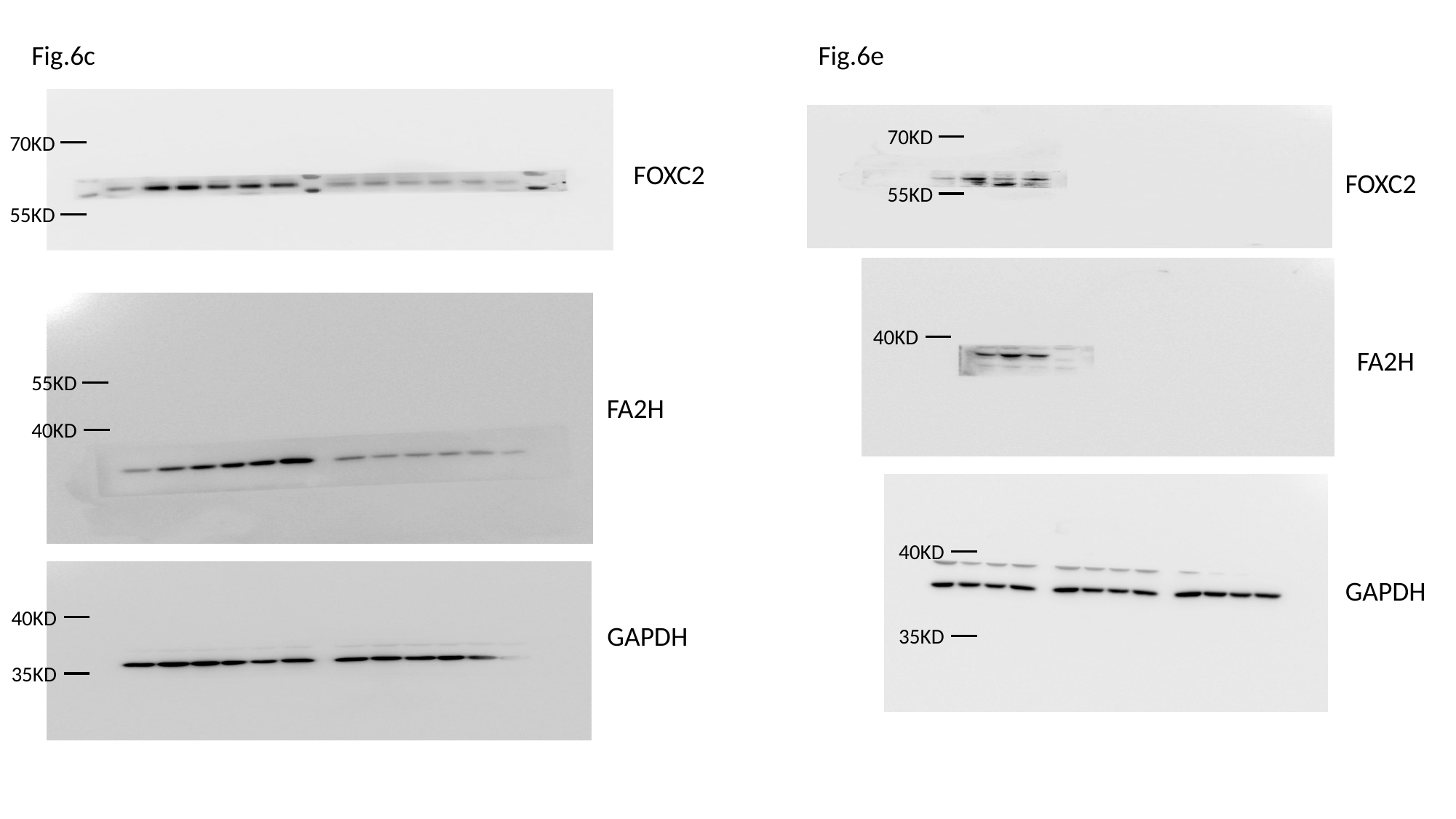

Fig.6c
Fig.6e
70KD
FOXC2
55KD
40KD
FA2H
40KD
GAPDH
35KD
70KD
FOXC2
55KD
55KD
FA2H
40KD
40KD
GAPDH
35KD

## Slide 6
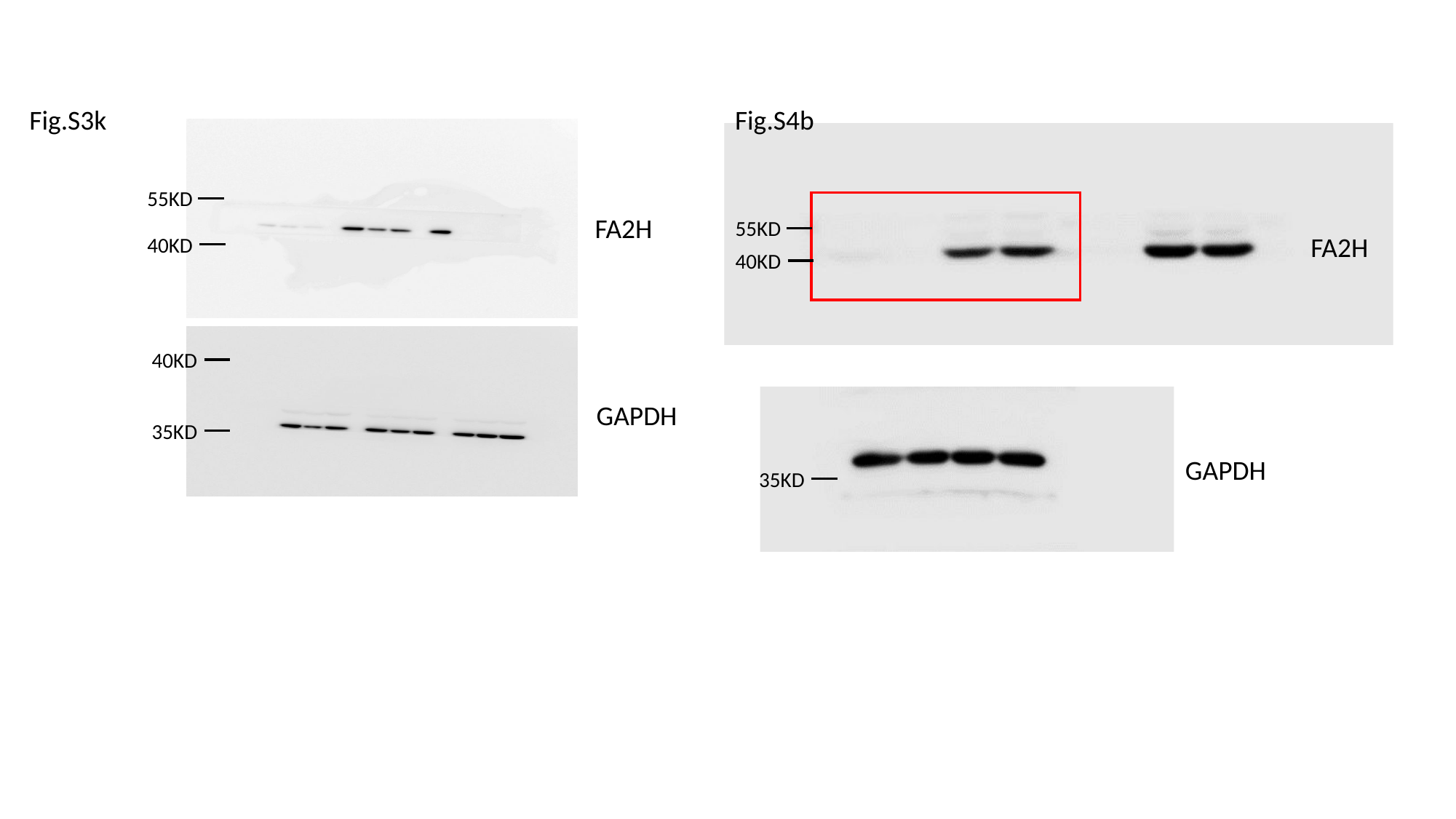

Fig.S3k
Fig.S4b
55KD
FA2H
40KD
40KD
GAPDH
35KD
55KD
FA2H
40KD
GAPDH
35KD

## Slide 7
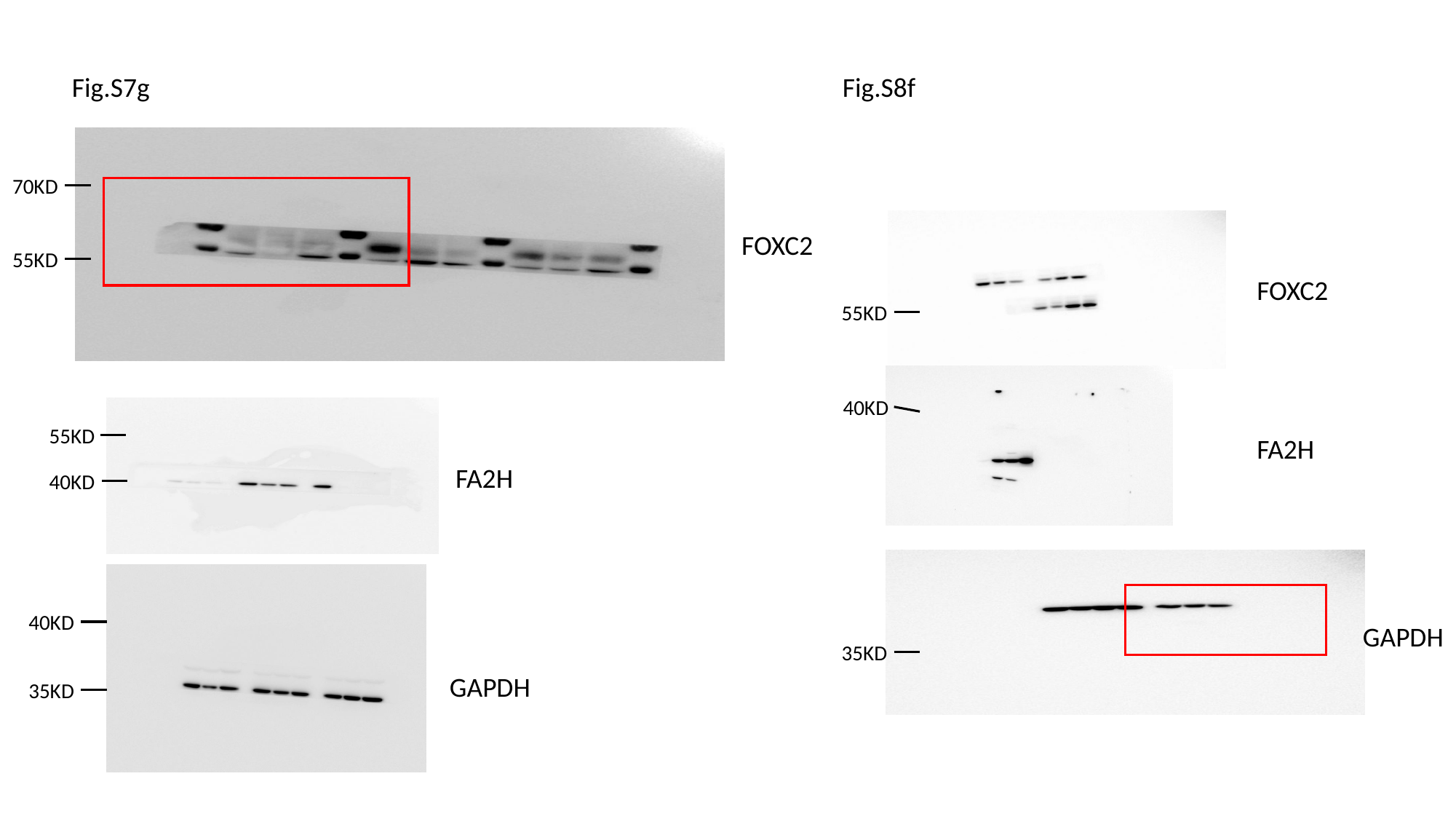

Fig.S7g
Fig.S8f
70KD
FOXC2
55KD
FOXC2
55KD
40KD
FA2H
GAPDH
35KD
55KD
FA2H
40KD
GAPDH
35KD
40KD

## Slide 8
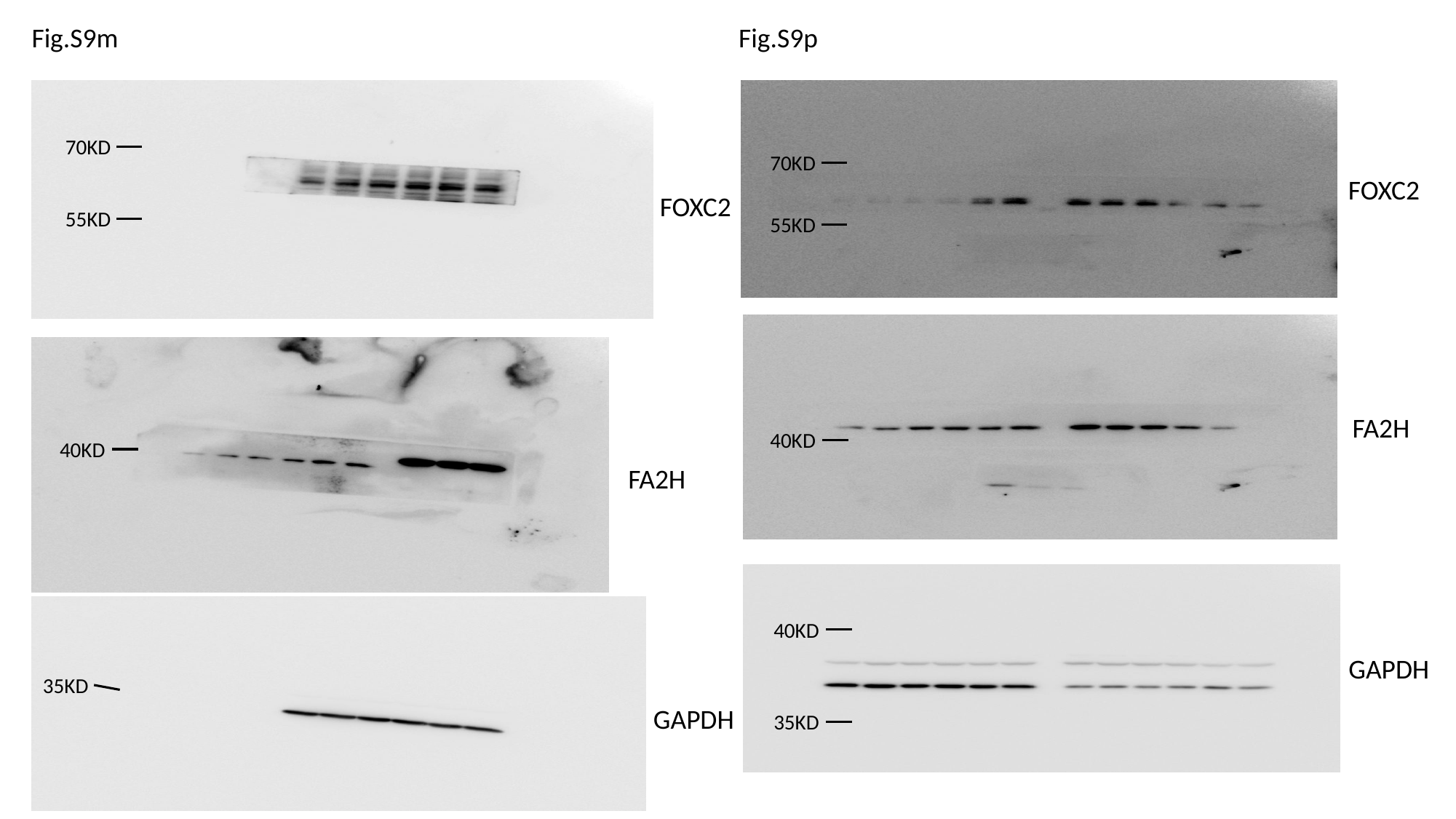

Fig.S9m
Fig.S9p
70KD
FOXC2
55KD
40KD
FA2H
35KD
GAPDH
70KD
FOXC2
55KD
FA2H
40KD
40KD
GAPDH
35KD

## Slide 9
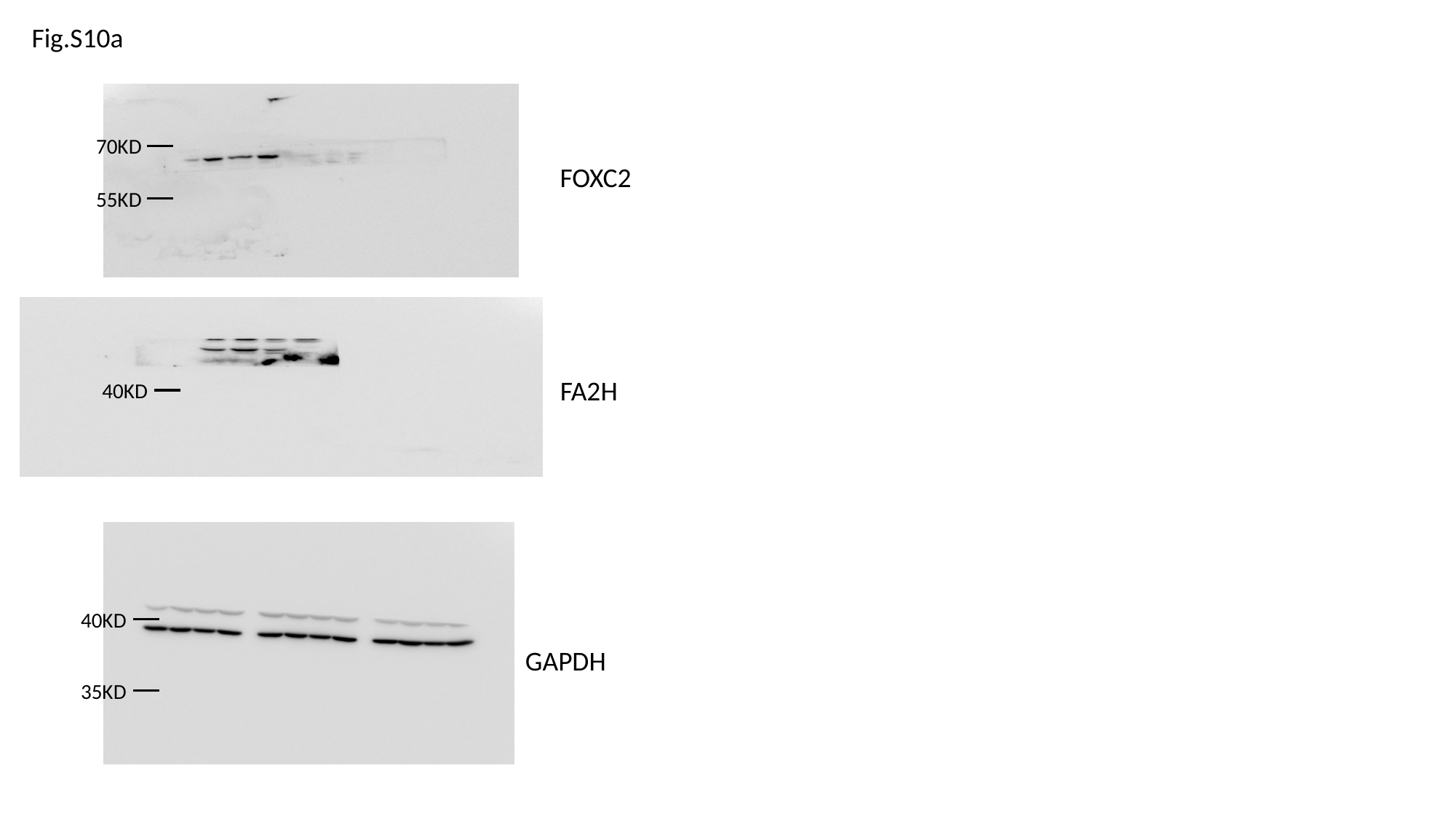

Fig.S10a
70KD
FOXC2
55KD
FA2H
40KD
40KD
GAPDH
35KD
