# Supplementary figures and images for: Correction: Dysregulated ceramides metabolism by fatty acid 2-hydroxylase exposes a metabolic vulnerability to target cancer metastasis
Source: Signal Transduct Target Ther. 2025 Sep 7;10:305. doi: 10.1038/s41392-025-02379-5 (PMC12415113; doi:10.1038/s41392-025-02379-5)

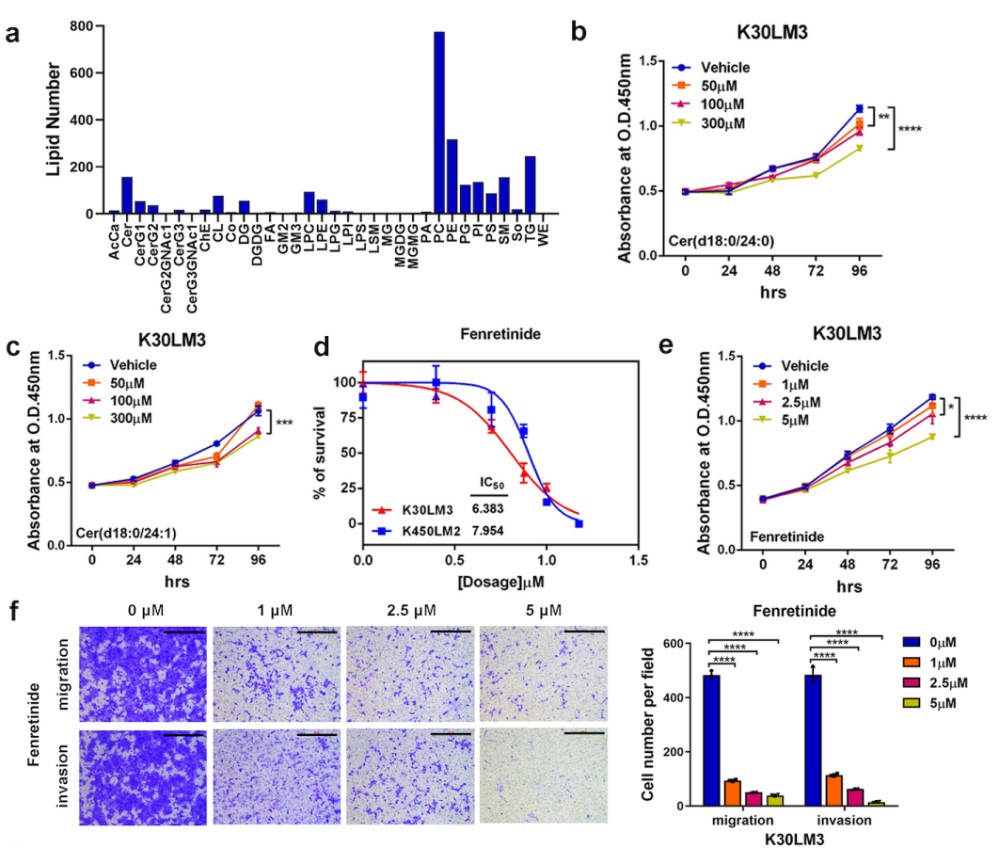

Supplement: Supplementary file 1 — Incorrect section of Figure S5f [file 41392_2025_2379_MOESM1_ESM.jpg]

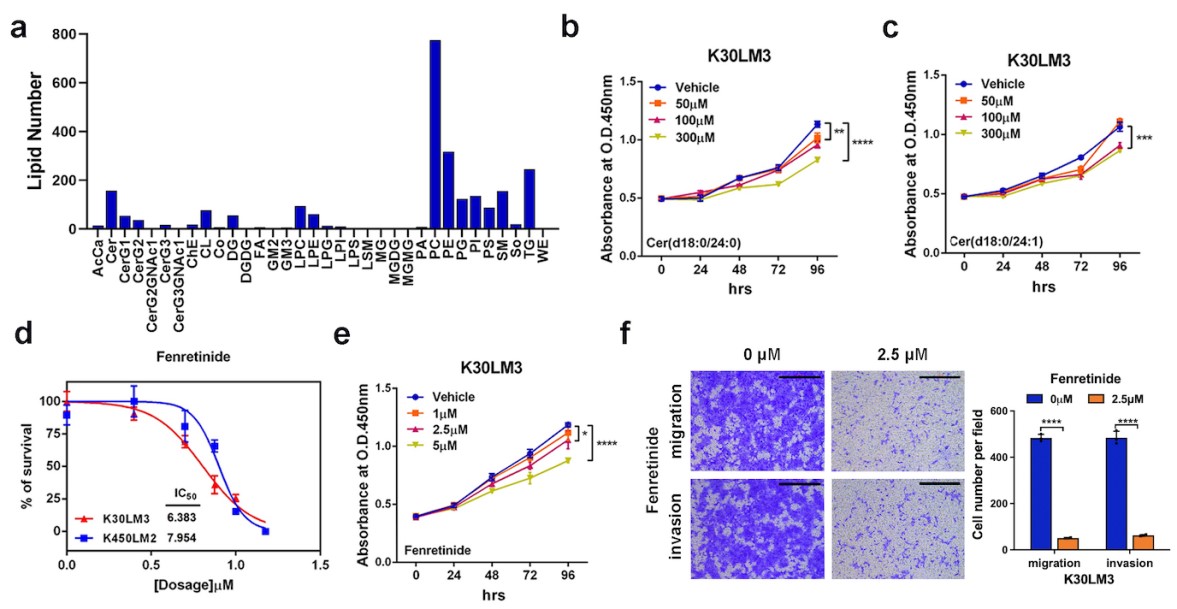

Supplement: Supplementary file 2 — Correct Figure S5f [file 41392_2025_2379_MOESM2_ESM.jpg]

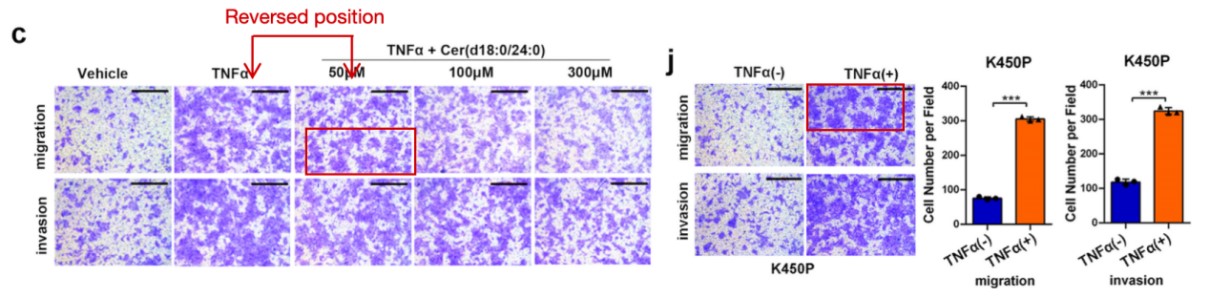

Supplement: Supplementary file 3 — Incorrect section of Fig. S10c and Fig. S9j [file 41392_2025_2379_MOESM3_ESM.jpg]

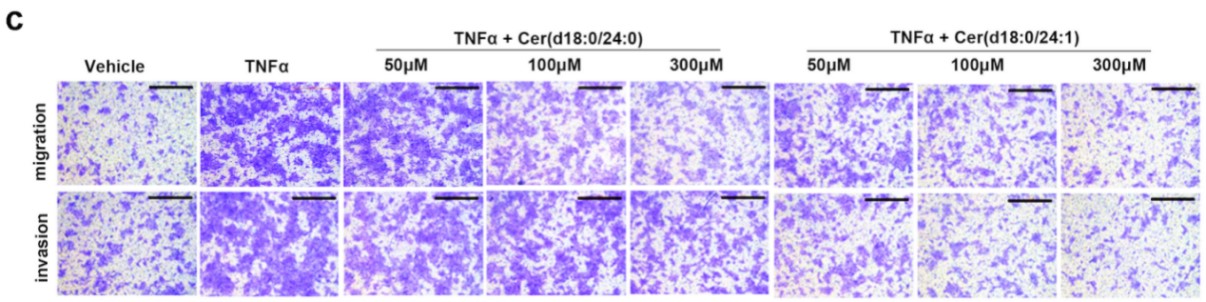

Supplement: Supplementary file 4 — Correct Figure S10c [file 41392_2025_2379_MOESM4_ESM.jpg]
